# Discovery and characterization of ORM‐11372, a novel inhibitor of the sodium‐calcium exchanger with positive inotropic activity

**DOI:** 10.1111/bph.15257

**Published:** 2020-11-10

**Authors:** Leena Otsomaa, Jouko Levijoki, Gerd Wohlfahrt, Hugh Chapman, Ari‐Pekka Koivisto, Kaisa Syrjänen, Tuula Koskelainen, Saara‐Elisa Peltokorpi, Piet Finckenberg, Aira Heikkilä, Najah Abi‐Gerges, Andre Ghetti, Paul E. Miller, Guy Page, Eero Mervaala, Norbert Nagy, Zsófia Kohajda, Norbert Jost, László Virág, András Varró, Julius Gy. Papp

**Affiliations:** ^1^ Orion Pharma, R&D Espoo Finland; ^2^ Department of Pharmacology Faculty of Medicine Helsinki Finland; ^3^ R&D AnaBios Corporation San Diego CA USA; ^4^ MTA‐SZTE Research Group of Cardiovascular Pharmacology Hungarian Academy of Sciences Szeged Hungary; ^5^ Department of Pharmacology and Pharmacotherapy, Interdisciplinary Excellence Centre, Faculty of Medicine University of Szeged Szeged Hungary

**Keywords:** cardiac safety, NCX, ORM‐11372, positive inotropic effect, sodium–calcium exchanger

## Abstract

**Background and Purpose:**

The lack of selective sodium–calcium exchanger (NCX) inhibitors has hampered the exploration of physiological and pathophysiological roles of cardiac NCX 1.1. We aimed to discover more potent and selective drug like NCX 1.1 inhibitor.

**Experimental Approach:**

A flavan series‐based pharmacophore model was constructed. Virtual screening helped us identify a novel scaffold for NCX inhibition. A distinctively different NCX 1.1 inhibitor, ORM‐11372, was discovered after lead optimization. Its potency against human and rat NCX 1.1 and selectivity against other ion channels was assessed. The cardiovascular effects of ORM‐11372 were studied in normal and infarcted rats and rabbits. Human cardiac safety was studied ex vivo using human ventricular trabeculae.

**Key Results:**

ORM‐11372 inhibited human NCX 1.1 reverse and forward currents; IC_50_ values were 5 and 6 nM respectively. ORM‐11372 inhibited human cardiac sodium 1.5 (*I*_Na_) and hERG K_V_11.1 currents (*I*_hERG_) in a concentration‐dependent manner; IC_50_ values were 23.2 and 10.0 μM. ORM‐11372 caused no changes in action potential duration; short‐term variability and triangulation were observed for concentrations of up to 10 μM. ORM‐11372 induced positive inotropic effects of 18 ± 6% and 35 ± 8% in anaesthetized rats with myocardial infarctions and in healthy rabbits respectively; no other haemodynamic effects were observed, except improved relaxation at the lowest dose.

**Conclusion and Implications:**

ORM‐11372, a unique, novel, and potent inhibitor of human and rat NCX 1.1, is a positive inotropic compound. NCX inhibition can induce clinically relevant improvements in left ventricular contractions without affecting relaxation, heart rate, or BP, without pro‐arrhythmic risk.

**What is already known**

- NCX plays a pathological role in heart failure, cardiac ischaemia, and arrhythmia.
- Known NCX modulators are unselective small molecules or peptides.

**What this study adds**

- The new NCX inhibitor ORM‐11372 was the most potent and selective inhibtor described so far.
- ORM‐11372 exerted positive inotropic effects in rabbits, without pro‐arrhythmic risk in human cardiac tissue.

**What is the clinical significance**

- ORM‐11372 exerted positive inotropic effect without other haemodynamic effects.

**Abbreviations**
*A*_max_: maximum amplitude of action potential
AP: action potential
APD_90_: action potential duration 90%
clogP: calculated logarithm of partition coefficient
clogS: calculated logarithm of solubility
CM: cardiomyocyte
ECC: excitation–contraction coupling
FCCP: carbonyl cyanide 4‐(trifluoromethoxyl)
HAM F‐12: Ham's Nutrient Mixture F‐12
HIPAA: Health Insurance Portability and Accountability Act
hiPSC: human‐induced pluripotent stem cell
hSCN5: human sodium voltage‐gated channel subunit 5
*I*_CaL_: L‐type Ca^2+^ channel current
*I*_hERG_: human ether‐á‐go‐go‐related gene‐encoded voltage‐dependent potassium channel current
IMR‐32: human neuroblastoma cell line
*I*_Na_: human cardiac Na_V_1.5 channel current
*I*_NCX_: Na^+^/Ca^2+^ exchanger current
IRB: institutional review board
LQT: long QT time
LV + *dP*/*dt*_max_: left ventricular inotropic effect
LV − *dP*/*dt*_min_: left ventricular relaxation
LVP: left ventricular pressure
*m*: *meta* position
MEM: minimum essential medium
MI: myocardial infarction
MRSA: methicillin‐resistant *Staphylococcus aureus*
NC3Rs: National Centre for the Replacement, Refinement & Reduction of Animals in Research
NCX_IF_: intrinsic factor‐inhibiting NCX
NIH: National Institutes of Health
PMCA: plasma membrane Ca^2+^ ATPase
RMP: resting membrane potential
SAR: structure–activity relationship
Sf9: insect cell *Spodoptera frugiperda*
SKCa: small‐conductance calcium‐dependent potassium channel
SP: systemic BP
STV: short‐term variability analysis of action potential duration
TNM‐FH: insect cell culture medium
XIP: exchanger‐inhibiting peptide

## 1 INTRODUCTION

The sodium–calcium exchangers (NCX; SLC8) play dynamic roles in excitation–contraction coupling (ECC) in cardiomyocytes (CMs). The driving force of NCX depends on sodium and calcium concentrations across the cell membrane cell as, well as the membrane potential. NCX operates dominantly in the forward mode (Ca^2+^ extrusion and inducing depolarizing current) during systole in all species. Therefore, selective NCX inhibitors have only minor effect on peak [Ca^2+^]_i_ in mice and dogs (Kohajda et al., 2016; Kormos et al., 2014; Oravecz et al., 2018). In addition, both mechanical relaxation and [Ca^2+^]_i_ decay remained unchanged (Kormos et al., 2014). Selective NCX inhibitors either slightly shorten action potential (AP) duration or have no effect in normal oxygen and ionic conditions. Overall, it seems that selective inhibition of NCX exerts minimal effecst on intracellular Ca^2+^ or action potential duration (APD) under normal conditions.

In heart failure, the intracellular Ca^2+^ concentration balance is changed, and the role of NCX becomes even more important (Bers & Despa, 2006). In addition, NCX and intracellular Ca^2+^ regulate each other and affect cardiac remodelling, as recently described (Primessnig et al., 2019). ORM‐11035, a selective NCX inhibitor, attenuated cardiac hypertrophic remodelling and prevented cardiac dysfunction in rats exhibiting heart failure. NCX also reportedly controls the heart rate (HR) through its effects on the sinus and atrioventricular nodes (Kaese et al., 2017). In sinus node, the funny current (*I*_f_) and the NCX current (*I*_NCX_) together establish a strong depolarization capacity providing an important safety factor for stable pacemaking (Kohajda et al., 2019). NCX also plays a role in BP control in normotensive and hypertensive individuals (Zhang, 2013).

The solute carrier transporter gene family (SLC8) encodes several Na^+^/Ca^2+^ exchanger subtypes. SLC8A1 gene overexpression and NCX1.1 protein up‐regulation are linked to many pathological conditions that lead to reduced contractility and arrhythmias (Khananshvili, 2013). NCX is organized into 10 transmembrane segments and is localized in the sarcolemmal membrane (Jost et al., 2013; Shattock et al., 2015). Two Ca^2+^ binding domains are known currently. Their activation is regulated by Ca^2+^ binding at these sites, whereas Na^+^ binding leads to NCX inactivation (Hilgemann, Matsuoka, Nagel, & Collins, 1992).

Several potent NCX inhibitors have been reported (Table S1). In 1996, KB‐R7943 was the first NCX inhibitor to be discovered. SN‐6 (Iwamoto et al., 2004) and SEA0400 (Matsuda et al., 2001) were reported to be more selective NCX inhibitors than KB‐R7943. YM‐244769 (Iwamoto & Kita, 2006), which was discovered in 2006, was reported to be a novel NCX inhibitor with higher selectivity. ORM‐10103 (Koskelainen, et al., 2003) was discovered early, followed by ORM‐10962 (Otsomaa, et al., 2004) and GYKB‐6635 (Geramipour et al., 2016) and all three were highly selective NCX inhibitors (Jost et al., 2013; Kohajda et al., 2016). Despite their higher selectivity, their use as positive inotropic tool compounds in experiments in vivo was prevented by their poor solubility (solubility of SEA0400 is less than 10 μg ml^‐1^ in pH 7.4 phosphate buffer which places it in the solubility class “insoluble”). ORM‐10962 is an exception from previously reported selective NCX inhibitors, as it exhibits reasonable solubility in in vivo studies.

Here, we describe the discovery of a new type of positive inotropic compound, ORM‐11372, exhibiting high selectivity for NCX1.1 and provide its pharmacological profile in vitro, in vivo*,* and ex‐vivo in human ventricular trabeculae. ORM‐11372 was developed for acute short‐term use with fast clearance.

## 2 METHODS

### 2.1 Discovery of a novel chemical series

**FIGURE 1:**
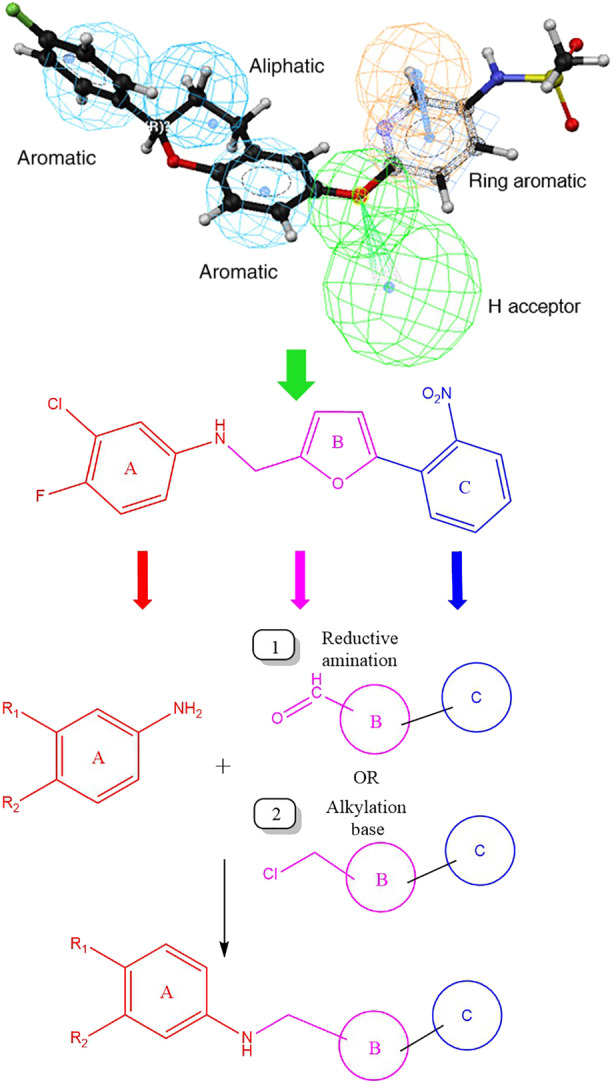
The upper part of the figure shows the pharmacophore model used for virtual screening. In silico hits were further filtered based on predicted activity, calculated logarithm of solubility (clogS), calculated logarithm of partition coefficient (clogP), and diversity. The middle part displays the hit structure and substructure features optimized in parallel processes. At the bottom of the figure, two routes used for the synthesis of the compounds are presented, namely, (1) reductive amination and (2) alkylation under basic conditions

**FIGURE 2:**
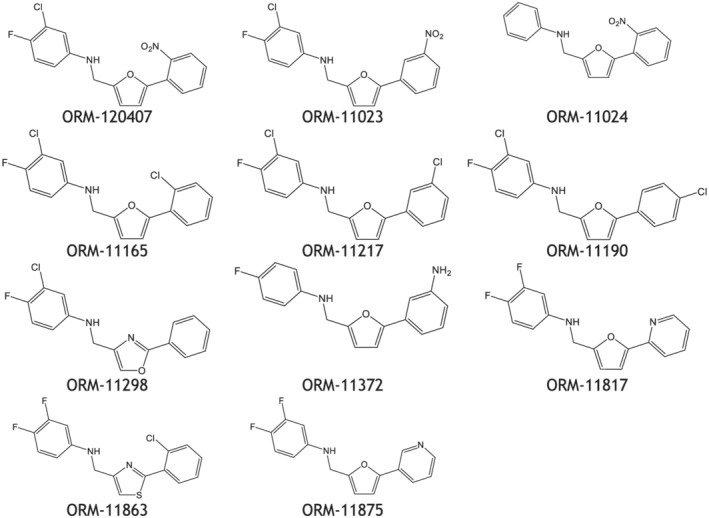
Illustrative chemical structures (out of 135 synthesized molecules) during the chemical optimization of the scaffold. ORM‐11372 had the most favourable profile overall, out of the synthesized derivatives

A novel and unique chemical series was discovered using ligand‐based pharmacophores for virtual screening with Catalyst (Accelrys) (Figure 1a). Pharmacophore features used for virtual screening were derived from previously discovered NCX1 inhibitor flavan structures, such as ORM‐10103 (Koskelainen et al., 2003) and ORM‐10962 (Otsomaa et al., 2004). Based on the results of virtual screening, a proposed library with 636 commercially available compounds was selected for testing in the fluorescence‐based assay, at concentrations of 10 μM. The original hit ORM‐120407 inhibited NCX1.1 by 87% and had an IC_50_ value of ~200 nM. It inhibited human ether‐á‐go‐go‐related gene (hERG) and L‐type Ca^2+^ channels, with IC_50_ values of 2.1 and 3.1 μM respectively. These results indicated that the scaffold exhibited optimization potential. The structure–activity relationship (SAR) of ORM‐120407 was explored further leading to the discovery of ORM‐11372 (Table 1 and Figure 2), was discovered during medicinal chemistry optimization.

**TABLE 1:** NCX inhibition values, as IC_50_, and the selectivity profile towards hERG and L‐type Ca^2+^ channels for selected compounds

Compound	NCX IC_50_ (nM)	hERG IC_50_ (μM)	L‐type Ca^2+^ IC_50_ (μM)	Solubility class
ORM‐120407	231	2.1	3.1	Insoluble
ORM‐11023	156	—	—	—
ORM‐11024	875	—	—	—
ORM‐11165	4	9	2	Insoluble
ORM‐11217	210	14.6	—	Moderate
ORM‐11190	204	—	—	—
ORM‐11298	31	>10	—	Moderate
ORM‐11372	6	10.6	6.1	Moderate
ORM‐11817	3	2.4	10.4	Insoluble
ORM‐11863	2.5	—	—	Insoluble
ORM‐11875	90	—	—	Moderate

The data shown are mean IC_50_ values from *n*=3 assays for NCX, *n*=4 assays for hERG and *n*=4 assays for L‐type Ca^2+^ channels. Each assay represents results from an independent plate. Abbreviations: hERG, human ether‐á‐go‐go‐related gene; *n*, number of independent plates; NCX, sodium–calcium exchanger.

An analysis of 250 previously discovered NCX inhibitor compounds by Orion (Koskelainen et al., 2003; Otsomaa et al., 2004) resulted in a five‐feature pharmacophore, which was used for the virtual screening of Cambridge and Specs compound libraries. In silico hits were further filtered based on predicted activity, calculated logarithm of solubility (clogS), calculated logarithm of partition coefficient (clogP), and diversity. The substructure features were optimized in parallel processes, and beneficial structural features were merged, in order to identify the potential overall synergistic effects for NCX1.1 inhibition. Representative samples of 135 synthesized derivatives are presented in Figure 2.

The hydrogen bond donor property of aniline in the original hit molecule ORM‐120407 was proven to be important for NCX1.1 activity in the scaffold. The addition of polar substituents was not tolerated in the A ring, but halogen substitution on the *p* and *m* positions was tolerated and resulted in improved NCX1 inhibition. However, neither position was favoured over the other nor showed synergistic or additive effects. Their selectivity towards the hERG channel was found to be its differentiating property.

SAR tolerated five‐membered B‐ring systems better than six‐membered systems. Ring heteroatoms, that is, oxygen or nitrogen molecules at position 1 (of furan) in combination with a 1,5‐substitution, were proven to be critical for binding; that is, a carbon at position 1 abolished activity. This finding indicates that the presence of a hydrogen bond acceptor at that position is important. B‐ring optimization provided three potential ring systems that were tolerated: furans, oxazoles, and thiazoles. Two latter systems tolerated 4,2‐substitution and 2,4‐substitution, while the three heteroatoms in the B‐ring reduced NCX1 inhibition activity. Though ORM‐120407 showed good inhibitory activity towards NCX1, its selectivity towards hERG (2.1 μM) and L‐type calcium (3.1 μM) channels needed to be improved. ORM‐120407 also had poor solubility (<10 μg·ml^−1^). ORM‐11298 exhibited better inhibitory activity towards NCX1 and good selectivity towards hERG channels. The oxazole series also exhibited good selectivity towards L‐type calcium channels such as ORM‐11298, but many derivatives were chemically unstable. The substitution of the C‐ring phenyl was unnecessary, but some substituents such as aniline and chlorine were tolerated. The replacement of the phenyl ring with different heteroaromatic ring systems was mainly tolerated.

### 2.2 Synthesis

Most compounds were prepared via reductive amination (Figure 1c, Route 1), except for ORM‐11298 and ORM‐11863, which were prepared by aniline alkylation (Figure 1c, Route 2). The reaction between the aniline and carbaldehyde derivatives could occur in the presence of a strong acid at elevated temperatures. The reduction of the imine intermediate could be carried out using a suitable reducing agent, such as sodium borohydride (NaBH_4_). The alkylation reaction in Route 2 is performed in the presence of a base. The products were isolated from the reaction mixture by extraction with ethyl acetate, followed by evaporation. The starting materials used in the processes were either commercially available or could be prepared via synthetic routes (Parry, Bryce, & Tarbit, 2003; Ye et al., 2010). ORM‐11372 was synthesized via the reductive amination of 5‐(3‐nitrophenyl)furan‐2‐carbaldehyde with the corresponding fluoroaniline, followed by the hydrogenation of the nitro group under mild conditions. Details of experiments can be found in Figures S1–S14.

### 2.3 Cell lines and cell culture

*Spodoptera frugiperda* (Sf9; RRID:CVCL_0549) cells are widely used for the transient and stable expression of recombinant proteins. Here, Sf9 cells were stably transfected with human NCX 1.1 and maintained in spinner flasks with insect cell culture medium (TNM‐FH), supplemented with 10% FBS, antibiotic–antimycotic solution, and 50 μg·ml^−1^ of blasticidin at 28°C in a non‐humidified, CO_2_‐free atmosphere. The Sf9 cell suspension was subcultured three times a week.

Human‐induced pluripotent stem cell (hiPSC)‐derived CMs (Cor.4U CMs; RRID:CVCL_Y550) were thawed, seeded onto gelatine‐coated coverslips, and maintained, as described in the manufacturer's protocol. Cor.4U cells express cardiac proteins including NCX1 and exhibit the relevant electrophysiology, demonstrating an ability to model human cardiac responses to drugs (Blinova et al., 2017; Huo et al., 2017).

CHO cells stably expressing either hERG1a (K_V_11.1) channels (KCNH2; RRID:CVCL_H512) or human 5‐HT_2B_ receptors were cultured at 37°C in a 5% CO_2_/95% air atmosphere in the Ham's Nutrient Mixture F‐12 (HAM F‐12) medium supplemented with 10% FBS (heat inactivated), 100 μg·ml^−1^ of hygromycin B (Invitrogen), and 100 μg·ml^−1^ of geneticin or 100 IU·ml^−1^ of penicillin and 100 IU·ml^−1^ of streptomycin, 25‐mM HEPES, 500 μg·ml^−1^ of geneticin, and 250 μg·ml^−1^ of Zeocin®. Adhered cells were detached using either Detachin® solution or trypsin and replated twice a week. HEK 293 (RRID:CVCL_0045) and IMR‐32 cells (CCL‐127; RRID:CVCL_0346) were cultured similarly but maintained instead in DMEM, supplemented with 10% FBS (heat inactivated), 100 IU·ml^−1^ of penicillin and 100 IU·ml^−1^ of streptomycin, and 25‐mM HEPES, and in the case of the IMR‐32 cell line, with minimum essential medium (MEM) non‐essential amino acids. IMR‐32 cells are derived from a human neuroblastoma and endogenously express L‐type (CaV1.x) calcium channel (Sher, Gotti, Pandiella, Madeddu, & Clementi, 1988).

The hERG‐encoded voltage‐dependent potassium channel current (*I*_hERG_) cells to be studied were harvested with Detachin solution and either diluted with a volume of CHO cell serum‐free media (supplemented with 100 IU·ml^−1^ of penicillin/streptomycin and 25‐mM HEPES) to obtain cells, for which the cell density was ~4 x10^6^ cells·ml^−1^, or plated on glass coverslips and used on the following 1–2 days. HEK 293 cells were transiently transfected using lipofectamine (Invitrogen, USA), with a human sodium voltage‐gated channel subunit 5 (hSCN5) A (transcript variant 2)‐containing plasmid, in Optimem medium. After a 5‐h incubation period, the transfection medium was replaced with the normal growth medium. The cells were harvested by trypsinization, centrifuged and resuspended in the appropriate extracellular solution for performing studies the next day. CHO–5‐HT_2B_ cells were plated into 96‐well plates on the previous day, at a density of 4 x10^4^ cells per well, with a modified version of the growth media.

### 2.4 Rat ventricular CMs

#### 2.4.1 Animals

All animal care and experimental procedures complied with the Guide for the Care and Use of Laboratory Animals (USA NIH publication No 85–23, revised 1996), and were approved by the Csongrád County Governmental Office for Food Safety and Animal Health, Hungary (approval No.: XIII/1211/2012). Animal studies are reported in compliance with the ARRIVE guidelines (Percie du Sert et al., 2020) and with the recommendations made by the British Journal of Pharmacology (Lilley et al., 2020). Six‐week old male Wistar rats (200‐250; RRID:RGD_13508588, obtained from a licensed supplier Toxi‐coop Ltd. Hungary) were used in this study.

#### 2.4.2 Housing and husbandry

The rats were maintained in standard rat cages (380 × 270 × 200 mm; ~1,025 cm^2^). The number of cage companions was four animals per cage. Cages were equipped with external bottle top‐type lids with half‐pocket wire bar lid feeders. The bedding of the cage floor was composed of aspen chips (Innovo Ltd., Hungary). The room temperature of the animal house was kept constant at 23°C, with a humidity of 40–65%. Twelve hours of dark–light cycle was applied with a low light intensity. Food (obtained from Innovo Ltd., Hungary) and tap water were provided ad libitum to the animals. The tap water is regularly checked for any pathogens.

#### 2.4.3 Cell preparation

Rats were anaesthetized with sodium thiopental (0.1 g·kg^−1^, i.p.) and injected with heparin sodium (500 IU, i.v.). Hearts were rapidly excised, mounted via the aorta on a Langendorff apparatus, and retrogradely perfused at 37°C with the Krebs–Henseleit solution for 5 min, with Krebs‐Henseleit solution (composition (in mM): 118.5 NaCl, 4 KCI, 2 CaCl_2_, 1 MgSO_4_, 1.2 NaH_2_PO_4_, 25 NaHCO_3_, and 11.1 glucose), pH 7.4 when saturated with a mixture of 95% O_2_ and 5% CO_2_. The perfusion of the heart was continued using Ca^2+^‐free Krebs–Henseleit solution for 10 min, and completed by the addition of 0.05% collagenase (type I), 0.05% hyaluronidase, and 200‐μM CaCl_2_, for a further 10 min. Subsequently, the left ventricular myocardium was minced and gently agitated. Dissociated cells were stored at room temperature in a solution containing (in mM) 89 KOH, 70 glutamate, 15 taurine, 30 KCI, 10 KH_2_PO_4_,10 HEPES, 0.5 MgCl_2_, 11 glucose, and 0.5 EGTA, and the pH was set to 7.3 using KOH. Cardiomyocytes were rod shaped and showed a clear striation, when external calcium levels were restored. One drop of the cell suspension was placed in a recording chamber and mounted on the stage of an inverted microscope (Olympus IX51, Olympus, Japan), and the individual myocytes were allowed to settle and adhere to the bottom of the chamber for at least 5 min before initiating superfusion.

### 2.5 Fluorescence screening assay for hNCX 1.1 inhibition

NCX reverse mode activity was stimulated via a 50% dilution of the extracellular Na^+^ level using the internal pipettor of FLEXstation, a fluorescence imaging plate reader (Molecular Devices, USA), while simultaneously monitoring intracellular calcium levels. After dilution, the concentration of other ions remained the same, but Na^+^ concentration was diluted to the half of original concentration of 172 to 86 mM. For Sf9 insect cells, normal extracellular Na^+^ ion concentration is 172 mM. Typically, 50 μl of a solution containing 150,000–200,000 Sf9–NCX cells was loaded onto each well of a 96‐well plate. Cells were preincubated with the intracellular calcium dye Fluo‐4 and Fluo‐6, and the concentrations of the test compound (three replicates) were determined for about 1 h at room temperature before experimentation. The extracellular solution contained (in mM) 172 NaCl, 10 HEPES, 1 CaCl_2_, 1.2 MgCl_2_, 0.33 NaH_2_PO_4_, 5 glucose, and 5 probenecid. The pH was adjusted to 7.4 with NaOH. The extracellular Na^+^ was diluted by the addition of a Na^+^‐free extracellular solution (containing in mM: 147 N‐methyl‐D‐glucamine (NMDG), 10 HEPES, 1 CaCl_2_, 1.2 MgCl_2_, 0.33 NaH_2_PO_4_, and 5 glucose with the pH adjusted by HCl to 7.4)‐induced robust and reproducible elevation of intracellular calcium. The osmolarity of both solutions was adjusted to 340–355 mOsm in order to match the Sf9 cell media osmolarity. In each plate, the IC_50_ determination for each test compound was based on relative fluorescence changes in comparison with control and 5‐mM nickel acetate‐induced NCX inhibition.

### 2.6 Confirmatory fluorescence assay for hNCX 1 inhibition

A confirmatory hNCX1 inhibition assay was performed in Charles River Laboratories, Cleveland. Briefly, HEK293 cells stably expressing hNCX1 were plated in 384‐well black wall, clear‐bottom microtitre plates and the next day loaded with Fluo‐8 for 30 min at 37°C. A plate was inserted into a FLIPR^TETRA^, and a baseline was recorded during a preincubation period where test compound or vehicle in a Na^+^‐free HB‐PS containing thapsigargin (6 μM) and carbonyl cyanide 4‐(trifluoromethoxyl) (FCCP) (30 μM) was added to each well for ~5 min. Next, the NCX1 stimulation period was recorded in which Na^+^‐containing HB‐PS (with 2‐μM thapsigargin and 10‐μM FCCP) was added. The experiments were performed at room temperature. The kinetic data generated were reduced for each well to maximum relative fluorescence units (RFU) minus minimum RFU after subtracting bias based on the first sample. The mean of the max–min RFU values during the stimulation period for the 3–4 replicates at each concentration on a plate was then plotted versus concentration and fitted to a Hill equation.

### 2.7 Fluorescence secondary screening assays

#### 2.7.1 L‐type calcium channel inhibition

Undifferentiated IMR‐32 cells were harvested, centrifuged, and resuspended in a probenecid‐Ringer solution consisting (in mM) 150 NaCl, 3 KCl, 1.2 MgCl_2_, 1 CaCl_2_, 20 HEPES, 5 glucose and 2.5 probenecid (pH 7.4 adjusted with NaOH, osmolarity 320–324 mOsm). We added 0.04% Pluronic F‐127 and Fluo‐4 to this solution, and after incubating the cells for 30 min at room temperature, these additives were removed from the probenecid‐Ringer solution by centrifugation and resuspension. The cell suspension was pipetted (250,000 cells in 75 μl per well) into a 96‐well plate, into which the test compound (75 μl per well at 2,667 × the final concentration; five concentrations with eight replicates) had already been added. The plate was centrifuged once (5 min, at 287 x *g*) to ensure that the cells were moved to the bottom of the wells. FLEXstation was then used to measure the increase in intracellular calcium at 37°C, following depolarization, which was induced by the addition (50 μl per well) of a KCl‐Ringer solution containing (in mM) 200 KCl, 20 CaCl_2_, 1.2 MgCl_2_, 20 HEPES and 2.5 probenecid respectively (pH adjusted to 7.4 with KOH, with an osmolarity of 320–324 mOsm).

#### 2.7.2 5‐HT_2B_ receptors

Changes in the intracellular calcium concentration of CHO–5‐HT_2B_ cells in the probenecid‐R inger were measured, using Fluo‐4 or calcium 3 dyes and the FLEXstation, at 37°C. ORM‐11372 was applied at seven concentrations (four replicates), 10 s after the initiating the measurement process in the agonist assay, but was preincubated (at eight concentrations with three replicates) in the antagonist assay, with 10‐nM 5‐HT added at the 10‐s time point.

### 2.8 Electrophysiology

Manual patch‐clamp recordings of membrane currents (*I*_NCX_, L‐type Ca^2+^ channel current [*I*_CaL_], human cardiac sodium 1.5 current [*I*_Na_], and *I*_hERG_) were undertaken using either an Axopatch 200B or Axopatch ID amplifier (Molecular Devices, USA), using the whole‐cell configuration. Cells were at least initially (except when recording *I*_Na_) perfused with an extracellular solution consisting of (in mM) 143 NaCl, 4 KCl, 1.8 CaCl_2_, 1.2 MgCl_2_, 5 glucose, and 10 HEPES (pH 7.4 with NaOH; osmolarity adjusted to 301 ± 3 mOsm). A slightly modified version was used for the rat ventricular CMs (in mM: 144 NaCl, 0.4 NaH_2_PO_4_, 4.0 KCl, 1.8 CaCl_2_, 0.53 MgSO_4_, 5.5 glucose, and 5.0 HEPES). Patch pipettes were pulled with a P‐2000 or P‐97 micropipette puller (Sutter Instruments, USA) from borosilicate glass capillaries and had resistances of between 2 and 4 MΩ when filled with any of the pipette solutions (see below). The data were filtered at 1 or 2 kHz and digitized at 10 kHz with acquisition and analysis performed by use of pClamp software (Versions 8–10; Molecular Devices; RRID:SCR_011323).

#### 2.8.1 NCX current (*I*_NCX_)

After the establishment of the whole‐cell configuration with hiPSC‐derived CMs or rat ventricular myocytes, the extracellular solution was switched to a K^+^‐free bath solution as described earlier (Hobai, Khananshvili, & Levi, 1997; Jost et al., 2013). The solution was composed of (in mM) 135 NaCl, 10 CsCl, 1 CaCl_2_, 1 MgCl_2_, 0.2 BaCl_2_, 0.33 NaH_2_PO_4_, 10 TEACI, 10 HEPES, and 10 glucose supplemented with 20‐μM ouabain, 50‐μM lidocaine, and 1‐μM nisoldipine or 2‐μM nitrendipine at pH 7.0 (osmolarity adjusted to 301 ± 3 mOsm). The pipette solution contained (in mM) 140 CsOH, 75 aspartic acid, 20 TEACI, 5 MgATP, 10 HEPES, 20 NaCl, 20 EGTA, and 10 CaCl_2_ (pH adjusted to 7.2 with CsOH; osmolarity adjusted to 290 ± 2 mOsm). The free intracellular Ca^2+^ level in the patch pipette was 140 nM according to Maxchelator (RRID:SCR_000459) software (Bers, Patton, & Nuccitelli, 2010).

The measurement of current in the K^+^‐free bath solution using a ramp voltage protocol at 20‐s intervals represented the first control, after which values were measured in the presence of one or more increasing concentrations of ORM‐11372 and finally upon exposure to 10‐mM NiCl_2_. The voltage protocol consisted of voltage ramps (at a rate of 100 mV·s^−1^) with a holding potential of −40 to 60 mV, which changed to −100 mV and then returned to −40 mV. *I*_NCX_ was defined as the Ni^2+^‐sensitive current value. However, for rat ventricular myocytes, the magnitude of the inward current (forward mode) was particularly small and variable. Therefore, to enhance the inward current and enable a solution with a certain concentration of ORM‐11372 to be measured, the concentrations of the K^+^‐free and pipette solutions were altered. In the bath solution, the CaCl_2_ concentration was reduced to 0.5 mM, while in the pipetted solution, the NaCl and EGTA concentrations were decreased to 5 and 10 mM respectively. All experiments were performed at 35–37°C. The outward human *I*_NCX_ inhibition current was measured at the following ORM‐11372 concentrations: 3 (*n* = 4), 10 (*n* = 6), 30 (*n* = 4), and 100 (*n* = 3) nM. The concentrations used for human inward *I*_NCX_ current measurement were 3 (*n* = 4), 10 (*n* = 4), 30 (*n* = 3), and 100 (*n* = 2) nM. The outward *I*_NCX_ current in rat CMs was measured at concentrations ranging from 1 to 1,000 nM (*n* = 5). The inward *I*_NCX_ current in rats was measured in additional experiments under changed conditions, to enhance the inward *I*_NCX_ current. In these experiments (*n* = 3), the effect of ORM‐11372 was tested at a concentration of 10 nM, at which the IC_50_ and reverse *I*_NCX_ current values were approximately equal.

#### 2.8.2 L‐type calcium current (*I*_CaL_)

*I*_CaL_ was recorded from hiPSC‐derived CMs (at room temperature, *n* = 2) and rat ventricular myocytes at 1 μM (*n* = 3; 5 cells) and at 10 μM (*n* = 4; 6 cells) in an extracellular solution supplemented with 4‐aminopyridine (3 mM) at 37^o^C. The pipette solution used for hiPSC‐derived CMs consisted (in mM) (pH 7.2; osmolarity adjusted to 293 mOsm) 110 KCl, 40 KOH, 20 TEACI, 3 MgATP, 10 EGTA, and 5 HEPES. The composition of the pipette solution used for rat CMs was (in mM) 125 CsCl, 20 TEACI, 5 MgATP, 10 EGTA, and 10 HEPES; the pH was adjusted to 7.2 using CsOH. *I*_CaL_ was evoked by a 400‐ms depolarization process to 0 mV from a holding potential of −40 mV every 5 s or, in the case of the rat CMs, by 400 ms; depolarizations to potentials ranging from −35 to 55 mV occurred after a prepulse to −40 mV, from the holding potential of −80 mV.

#### 2.8.3 Na_V_1.5 channel current (*I*_Na_)

Recordings were performed on HEK cells (*n* = 3) that transiently expressed voltage‐gated sodium channel alpha subunit 5 (SCN5A) at room temperature, in a bath solution containing (in mM) (pH 7.4 with NMDG; osmolarity adjusted to 300 ± 2 mOsm) of 40 NaCl, 97 l‐aspartic acid, 4 KCl, 1.8 CaCl_2_, 1 MgCl_2_, 10 glucose, and 10 HEPES. The pipette solution contained (pH 7.2 with CsOH; osmolarity adjusted to 270 ± 3 mOsm) 130 caesium methane sulfonate, 5 MgCl_2_, 5 EGTA, 0.1 GTP, 4 ATP disodium salt hydrate (Na_2_ATP), and 10 HEPES. The voltage protocol, which was repeated after each second, included of the process of hyperpolarization, from a holding potential of −80 to −120 mV for 200 ms, followed by that of depolarization to −15 mV for 10 ms. The peak current values observed while applying the test pulse at −15 mV were used for analysis.

#### 2.8.4 K_V_11.1 channel current (*I*_hERG_)

Values from hERG‐expressing cells (*n* = 4) were recorded with the standard extracellular solution at the physiological temperature and a pipette solution containing (in mM) 130 KCl, 7 NaCl, 5 EGTA, 1 MgCl_2_, 5 Na_2_ATP, and 5 HEPES (pH was set to 7.2 with KOH; osmolarity was adjusted to 290 ± 3 mOsm). *I*_hERG_ was evoked using a voltage protocol, which was repeatedly performed every 10 s, consisting of a depolarization step from the holding potential of −75 to 10 mV for 500 ms, followed by a repolarization step to −40 mV for 500 ms. The peak tail current values at −40 mV were used for analysis.

In addition, after loading the CHO–hERG cell suspension, whole‐cell voltage‐clamp values were recorded at room temperature on a QPatch 16× automated patch‐clamp (Sophion Biosciences), in the single‐hole mode. ORM‐11372 solutions with concentration of 0.3 (*n* = 4), 1 (*n* = 5), 3 (*n* = 8), 10 (*n* = 8), and 30 (*n* = 8) μM were studied. The extracellular solution contained (in mM) 145 NaCl, 4 KCl, 2 CaCl_2_, 1 MgCl_2_, 10 glucose, and 10 HEPES (pH 7.4 with NaOH; osmolarity adjusted to 305 mOsm). The intracellular recording solution contained (in mM) 120 KCl, 1.75 MgCl_2_, 5.37 CaCl_2_, 4 Na_2_ATP, 10 EGTA, and 10 HEPES (pH 7.2 with KOH; osmolarity adjusted to 295 mOsm). The voltage protocol, which was repeated every 10 s, included a 200‐ms step to change the holding potential from −80 to −50 mV, to measure the leak current, and further depolarization to +20 mV for 2 s, followed by a repolarization to −50 mV for 2 s. The hERG tail current was measured as the difference between the peak tail current amplitude during the repolarization step and leak current measurement step. Subsequently, the voltage dependence of the block was also assessed, by carrying out depolarization for 4 s, from −80 to +60 mV in 10‐mV steps, before and after the addition of 20‐μM ORM‐11372 (*n* = 7). The peak tail current elicited during the 5 s repolarization step to −50 mV was measured and plotted against the preceding depolarization step voltage values.

#### 2.8.5 Action potentials

APs were recorded in spontaneously beating Cor.4U CMs in the current clamp using the perforated patch technique and voltage‐sensitive dye (di‐4‐ANEPPS), in a 96‐well plate format. The latter ratiometric optical measurements were performed on the CellOPTIQ platform at Clyde Biosciences (Newhouse, UK). The patch‐clamp measurements used the standard extracellular solution at the physiological temperature and a pipette solution containing (in mM) 122 K‐gluconate, 30 KCl, 1 MgCl_2_, and 5 HEPES (pH 7.2 with KOH; osmolarity adjusted to 290 ± 3 mOsm) and 0.24 mg·ml^−1^ of amphotericin B. The patch‐clamp data shown are from CMs (*n* = 4) that exhibited ventricular‐like APs, that is, a ratio (time difference between APD_30_ and APD_40_/time difference between APD_70_ and APD_80_) >1.5 (Ma et al., 2011).

#### 2.8.6 Selectivity panel

Radioligand binding assays were performed at Eurofins Cerep SA (Celle L'Evescault, France), for testing the inhibition capacity of ORM‐11372 (at 10 μM with two replicates), in over 75 targets. These included receptors (e.g., human A_1_, A_2A_, and A_3_ adenosine receptors; α_1_ and α_2_ adrenoceptors; human β_1_–β_3_ adrenergic receptors; human D_1_, D_2S_, D_3_, D_4.4_, and D_5_ dopamine receptors; EGF and VEGF receptors; human M_1_–M_5_ muscarinic ACh receptors; human 5‐HT_1A_, 5‐HT_2A_, 5‐HT_2B_, 5‐HT_2C_, 5‐HT_4e_, 5‐HT_5A_, 5‐HT_6_, and 5‐HT_7_ receptors), ion channels (5‐HT_3_, Ca^2+^, ATP‐dependent potassium channel [K_ATP_], voltage‐dependent potassium channel [K_V_], small‐conductance calcium‐dependent potassium channel [SK_Ca_], Na^+^, and Cl^−^), and transporters (adenosine, noradrenaline, dopamine, and 5‐HT). Follow‐up functional studies (with five concentrations and two replicates) were performed for 5‐HT_2A_ receptors and noradrenaline transporter. Agonism and antagonism at h5‐HT_2A_ receptors was explored in recombinant HEK 293 cells and intracellular calcium changes were detected using fluorimetry. Noradrenaline uptake was measured by carrying out scintillation counting of [^3^H]noradrenaline incorporated into rat hypothalamus synaptosomes.

### 2.9 Design and analysis for isolated heart preparations and in vivo experiments

#### 2.9.1 Donor heart procurement

All the human hearts used for this study were obtained after legal consent and were provided by organ donors in the United States. The policies for donor screening and consent were the same as those established by the United Network for Organ Sharing (OPTN, 2019). Organizations supplying human tissues to AnaBios follow the standards and procedures established by the US Centers for Disease Control and Prevention and are inspected biannually by the Department of Health and Human Services. Tissue distribution is governed by internal institutional review board (IRB) procedures and was compliance with Health Insurance Portability and Accountability Act (HIPAA) regulations (Edemekong & Haydel, 2019) regarding patient privacy. All organ donor transfers to AnaBios are fully traceable and periodically reviewed by US federal authorities. In general, AnaBios obtains donor hearts from adults aged 17–60 years old. Though some donors were trauma victims, donors with the following conditions were excluded: ejection fraction <45%, HIV, cardiac death, hepatitis B virus (HBV), congenital long QT time (LQT) syndrome, hepatitis C virus (HCV), LOT syndrome, methicillin‐resistant *Staphylococcus aureus* (MRSA), downtime >20 min, ongoing infections, positive blood cultures without treatment, and 48‐h result data. Donor hearts from 1 male and 1 female (both 57 years old, Table S5) were harvested using AnaBios' proprietary surgical techniques and tools and were shipped to AnaBios via dedicated couriers. Upon arriving at AnaBios, each heart was assigned a unique identifier number that was reproduced on all relevant medical history files, data entry forms, and electronic records.

#### 2.9.2 APs in human ventricular trabeculae

Procedures used for tissue dissection and recording were similar to those described previously (Page et al., 2016). Briefly, the human heart was transferred into a dissection vessel containing a cold (4°C), fresh proprietary dissection solution. The heart was completely submerged into the dissection solution. Ventricular trabeculae were dissected and transferred to the recording chamber. The approach used to record APs is similar to that described by Page et al. (2016). Briefly, a single tissue was mounted into the experimental chamber filled with oxygenated Tyrode's external solution, containing (in mM) 136 NaCl, 4 KCl, 0.5 MgCl_2_, 12 NaHCO_3_, 0.35 NaH_2_PO_4_, 11.1 dextrose, 1.8 CaCl_2_, and 10 HEPES (pH 7.4). The temperature of the solution was maintained at 37°C, at a flow rate of 5 ml·min^−1^. The tissue was allowed to equilibrate for 30–60 min, while providing stimulation (3 V, 3 ms) at a frequency of 1.0 Hz. High‐impedance borosilicate microelectrodes were prepared with a tip resistance of 10–20 MΏ, filled with 3‐M KCl. Upon tissue impalement, the membrane potential was allowed to stabilize (typically, around −85 mV). Tissues with resting membrane potentials (RMPs) more positive than −75 mV were rejected. Bipolar stimulation at 1.5× threshold was applied, and recordings were obtained in the continuous mode with sampling at 20 kHz, using ADInstruments and LabChart software. Tissue exclusion criteria included the following: (i) interruption of perfusion/oxygenation; (ii) absence of APs following stimulation at baseline; (iii) time frame of drug exposure not respected; (iv) unstable response to stimulation at baseline; (v) RMP > −75 mV; (vi) maximal amplitude of AP (*A*_max_) < 70 mV; and (vii) AP duration at 90% repolarization (APD90) < 200 or >450 ms. ORM‐11372 was evaluated at three concentrations in four ventricular trabeculae derived from two donor hearts (*n* = 2, two replicates). Testing concentrations were 0.1, 1, and 10 mM. Following the stabilization of each tissue, APs were collected and assessed for 31 min in the vehicle control solution (Tyrode with 0.1% DMSO), at stimulation frequencies of 1 Hz for 25 min, 2 Hz for 3 min, and 1 Hz for 3 min. Following this vehicle control period, three concentrations of ORM‐11372 were applied sequentially and cumulatively. Each concentration was applied for 31 min with the same stimulation sequence as in the vehicle controls.

### 2.10 Design and analysis of animal experiments

All animal experiments were performed according to European Community Guidelines for the use of experimental animals and approved by the Finnish National Animal Experiment Board. Animal studies are reported in compliance with the ARRIVE guidelines (Percie du Sert et al., 2020) and with the recommendations made by the *British Journal of Pharmacology* (Lilley et al., 2020).

#### 2.10.1 Selection of animal species

The rat is the most widely used rodent species in toxicology studies performed for drug development. The Sprague–Dawley rat strain (RRID:RGD_70508) is commonly used in the rat myocardial infarction (MI) model (Fishbein, Maclean, & Maroko, 1978). However, the functional similarity between the Ca^2+^ handling proteins (including NCX) in rats (Bassani, Bassani, & Bers, 1994) and human hearts is rather low. The Ca^2+^ circulation balance in the guinea pig and rabbit hearts are more similar to that of a human (Milani‐Nejad & Janssen, 2014). Rabbits and humans are also known to react similarly to medication; hence, the rabbit was selected as a nonrodent species.

#### 2.10.2 Housing and husbandry

Animals were monitored daily by laboratory personnel. If the general health status of an animal was significantly worsened, the animal was killed with an overdose of pentobarbital. Human endpoints included no spontaneous movements and inability to drink or eat during the 24‐h observation period, massive bleeding, spontaneous inflammation, missing anatomical features or swelling, and breathing difficulties. Specific pathogen‐free animals were housed in half‐barrier rooms where special protective clothing was required by personnel. Animal rooms were cleaned regularly three times per week, and cages and bottles were changed at regular intervals once a week. The temperature was maintained at 22 ± 2°C and humidity at 55 ± 15%. In the light–dark cycle, lights were kept on from 6:00 a.m. to 8:00 p.m. Body weights of all animals were measured weekly.

#### 2.10.3 Randomization and blinding

Randomization was used whenever feasible. Guinea pigs were randomized into vehicle and ORM‐11372 groups for in vitro contraction force measurement from papillary muscle. In haemodynamic studies involving rabbits, only one study group was used, according to the 3R principle. Because the experimental set‐up involved ascending doses in one study group, randomization was impossible. Baseline values were used as controls to reduce variability. However, the MI rat model required the inclusion of two study groups, to eliminate the effect of surgical operations. The MI study set‐up enabled the use of randomization. The study duration for anaesthetized animals is limited; hence, higher doses had to be administered, which required blinding to be performed in all in vivo studies. The blinding was not considered relevant, because measured absolute values and derived parameters are not sensitive to biased interpretations.

### 2.11 In vitro contraction dynamics measurement

Guinea pigs (both sex, body weight ranging 342–565 g, Dunkin Hartley, M&B A/S, Ry, Denmark) were used in the study. The animals were housed in solid bottom polycarbonate cages (Makrolon® IV, 385 × 590 × 200 mm) with stainless steel wire mesh lids, up to three animals of same sex per cage. Autoclaved aspen chips (Tapvei Ky, Kaavi, Finland) were used as bedding. A commercially available rodent SDS FD1 (P) SQC pellet diet (Special Diet Services Ltd, Witham, England) and tap water from the public supply (Espoon vesilaitos, Espoo, Finland) were available ad libitum in polycarbonate bottles (800–1,000 ml) with plastic caps (Scanbur A/S, Ejby, Denmark).

Guinea pigs were killed by a blow on the skull and the heart will be excised. Right ventricular papillary muscle was dissected and rinsed in ice‐cold Tyrode's solution. Thereafter, the papillary muscle was mounted for the measurement of twitch tension in an organ bath containing modified Tyrode's solution (at 37°C) bubbled with carbogen (95% O_2_ and 5% CO_2_). The composition of Tyrode's solution was as follows (in mM): 135 NaCl, 1 MgCl_2_·6H_2_O, 5 KCl, 2 CaCl_2_·2H_2_O, 15 NaHCO_3_, 1 Na_2_HPO_4_·2H_2_O, and 10 glucose at pH 7.3–7.4.

The signal was acquired with a validated acquisition system ACFO v1.0 (Fision Oy, Finland). The following parameters were measured: twitch tension (Tw), rest tension (RT), time to peak tension (TTP), and half relaxation time (HRT). At steady state, 15 twitch tensions were acquired and averaged was used for statistics.

The baseline twitch tensions were measured after a stabilization period of 20–40 min. Thereafter, vehicle (1.1% DMSO) or ORM‐11372 in Tyrode's solution was superfused into the cuvette for 15 min, and the effects were measured from the steady‐state level. All experiments were carried out at 37°C.

### 2.12 Experimental MI model

Male Sprague–Dawley rats (Harlan, Netherlands B.V.) were housed in polycarbonate cages (Makrolon IV with stainless steel wire mesh lids). A maximum of five rats were housed per cage with aspen chip bedding (Tapvei Ky, Kaavi, Finland). Rats had free access to water (twice filtered, Espoon Vesi) and a rodent diet (SDS RM1 (P) SQC pelleted diet, Special Diet Services Ltd, Witham, England). The acclimation period before experiments was at least 5 days. In the MI model, rats (7–8 weeks, 200–250 g) were randomized into either the MI or sham group. In this weight range, a rat is considered a young adult, when its evolution phase is characterized by slow growth and its surgical mortality is lower than that of older animals. Male rats were used to minimize the variability of the cardiac response to several stimuli (Zornoff, Paiva, Minicucci, & Spadaro, 2009).

Rats were anaesthetized with a combination of ketamine (Ketalar® 50 mg·kg−1, i.p.) and medetomidine (Domitor 250 μg·kg^−1^, i.p.), intubated, and artificially ventilated with air (Ugo Basile 7025). An MI was induced by the occlusion of the left coronary artery under aseptic conditions (Levijoki, Pollesello, Kaheinen, & Haikala, 2001; Selye, Bajusz, Grasso, & Mendell, 1960). The left coronary artery was ligated at a distance of about 2–3 mm from the origin of the aorta with a silk suture, and the heart was repositioned into the chest. After ligation, the wound was closed carefully. The muscle layer was closed with soluble stitches and skin layers with insoluble stitches, followed by a partial reversal of anaesthesia via the intraperitoneal administration of atipamezole (Antisedan® 0.3–1 mg·kg^−1^, i.m.). The trachea tube was removed, and the trachea and underlying muscle and skin layers were closed immediately. To prevent dehydration, rats were subcutaneously administered with 5 ml of 0.9% NaCl. After surgery, rats were given analgesia with buprenorphine for at least 2 days (0.05 mg·kg^−1^, s.c. twice a day). For sham‐operated rats, the same procedure was performed, without ligation. Rats were analysed for 7 days after the MI, for signs of severe acute heart failure such as oedema or breathing difficulties.

### 2.13 Haemodynamic measurements of anaesthetized animals

All animals were given multiple ascending infusion doses of ORM‐11372; blinding was not possible. Systemic BP (SP) and left ventricular pressure (LVP) values were measured with pressure transducers (SP for femoral artery: Isotec, Hugo Sachs Elektronic, Germany; a miniature pressure transducer Mikro‐Tip transducer SPR‐249, Millar Instruments was inserted into the left ventricle via the right carotid artery). The signal was amplified (DC‐bridge amplifier type 660, Hugo Sachs Elektronik, Germany), digitized (I/O connector block type SCB‐68, National Instruments, USA), recorded, and analysed (IHME 1.0.9, Fision Ltd, Finland). Left ventricular inotropic effect (LV + *dP*/*dt*_max_), relaxation (LV − *dP*/*dt*_min_), and HR values were analysed from LVP signals.

At the end of the haemodynamic experiments, rats and rabbits were killed with an overdose of pentobarbital. Small laboratory equipment (forceps, scissors, scalpels, etc.) were sterilized in the glass bead sterilizer at 300°C for 10 seconds.

The BP waveform was measured continuously in milliseconds. Blinding was not considered relevant, because the absolute value was not sensitive to biased interpretations, and all parameters are derived from of them.

#### 2.13.1 Haemodynamics in MI rats

Haemodynamics were assessed 7 days after the MI (*n* = 6) or sham operation (*n* = 6) as follows. Rats were anaesthetized with isoflurane (2.25–2.5%, Baxter) in carbogen (95% O_2_ and 5% CO_2_) and nitrous oxide (1:1), using a small rodent ventilator (Ugo Basile 7025, ~10 ml·kg^−1^, 60 strokes·min^−1^). The rats were infused with 0.9% NaCl in the carotid vein at the stabilization and baseline levels. ORM‐11372 was administered into MI (*n* = 6) and sham (*n* = 6) rats in ascending infusion doses of 1.7, 17, 167, and 417 μg·kg^−1^·min^−1^. Infusions were administered into the jugular vein of MI rats at the infusion rate of 5 ml·kg^−1^·h^−1^ (Terumo TE‐311, Belgium). Doses were selected based on a pilot study with healthy rats (Figure S20). The total number of animals in the study was 12 + 2, due to the replacement of two MI rats. One MI rat died while administering anaesthesia, as infarcted animals are sensitive to anaesthesia, and another MI rat was excluded due to the dosing error indicated by the bioanalysis of plasma samples (no ORM‐11372 concentration observed in plasma).

After haemodynamic assessments, the anaesthetized rats were killed with an overdose of pentobarbital (2 ml of Mebunat® vet 60 mg·ml^−1^ per rat). The entire heart of each animal was fixed in buffered 4% formaldehyde solution, trimmed, processed, and embedded in paraffin. The hearts were cut horizontally at three different levels: apex, mid part, and base. Sections (4 μm) were cut from each level stained with haematoxylin and eosin, for general histopathological analysis. Infarct sizes were determined using Picrosirius Red staining as an indicator of cardiac fibrosis, and the size was measured using AnalysisPro software. The infarct area was calculated as the per cent of fibrotic tissue in the total myocardial area (mean of three levels).

#### 2.13.2 Haemodynamics in rabbits

Male New Zealand white rabbits (Harlan, Netherlands B.V.) were housed individually in polycarbonate cages (Scanbur Number 8 plastic cages with stainless steel door) with aspen bedding (Aspen Bricks M, Tapvei Ky, Kaavi, Finland). The acclimation period before experiments was at least 21 days. Rabbits weighing 2.0–2.3 kg (age 10–12 weeks) were used for haemodynamically assessing ORM‐11372 (no randomization, baseline values as own control). The diet provided to rabbits was SDS Stanrab (P) SQC pelleted (Special Diet Services Ltd, Witham, England). Water was provided ad libitum in polycarbonate bottles (750 ml).

Rabbits (*n* = 5) were sedated with i.v. (marginal vein) diazepam (2 mg·kg^−1^, Diapam®, Orion Pharma). Anaesthesia was induced with i.v. S‐ketamine (10–20 mg·kg^−1^ Ketanest‐S®, Pfizer) and maintained with S‐ketamine i.v. infusion (15–80 mg·kg^−1^·h^−1^). Animals were placed on a heating table (+38°C), and tracheas were cannulated. Rabbits were ventilated via a rodent ventilator (Ugo Basile 7025, Hugo Sachs Elektronik, Germany; respiratory volume 10 ml·kg^−1^, 30 strokes·min^−1^ for rabbits). ORM‐11372 was infused into the jugular vein at an infusion rate of 10 ml·kg^−1^·h^−1^ (Terumo TE‐311, Belgium), at infusion doses of 17, 167, and 833 μg·kg^−1^·min^−1^.

### 2.14 Blood sampling for bioanalysis

At the end of each infusion process, blood samples (300–500 μl) were added to a chilled EDTA polypropylene tube (CapiJect®, Terumo) and centrifuged (1431 x *g*, 10 min, at 4°C). Plasma was immediately frozen in polypropylene tubes and stored at −20°C. ORM‐11372 was extracted from plasma samples using a liquid–liquid extraction process and analysed using LC–MS/MS (Agilent Technologies series 1100 LC system and a Sciex LC–MS/MS API 4000 MS). The lower limit of quantification for the plasma concentration of ORM‐11372 was 32.0 ng·ml^−1^. Plasma protein binding in ORM‐11372 was assessed using the TRANSIL High Sensitivity kit, as per the manufacturer's instructions (Sovicell, GmbH, Germany).

### 2.15 Data and statistical analysis

Results from the experiments with human trabeculae were expressed as APs at each selected frequency. For each frequency tested, the last 30 APs acquired at the end of the period were averaged for vehicle controls, for each ORM‐11372 solution with a certain concentration. Analysis at 1 Hz included only the last 30 APs from the initial 25 min incubation period. The following AP parameters and pro‐arrhythmia variables were analysed offline upon the completion of recordings: RMP (mV), *A*_MAX_ (mV), AP duration at percent repolarization (APD30, APD50, APD90) (ms), short term variability analysis of AP duration (STV), triangulation (APD90‐APD30).

STV was calculated as the beat‐to‐beat variability in repolarization from APD90 Poincare plots over a 30 sec duration. STV for APD90 was calculated as follows: STV = ⅀ |APDn+1−APDn|/(30×√2), where APD (*n*) and APD(*n*+1) are the APDs for the *n*th AP and the following AP respectively.

The effects of ORM‐11372 were quantified relative to the data collected during the vehicle control period. Threshold values for changes over the baseline control for APD30, APD50, APD90, triangulation, and STV at 1 and 2 Hz pacing frequencies have been determined in a previous validation study (Page et al., 2016). Results are expressed as mean ± SEM.

The data and statistical analysis of in vitro and in vivo studies comply with the recommendations for experimental design and analysis in pharmacology (Curtis et al., 2018). Data were analysed statistically only when the number of independent samples was 5 or more. A P‐value of <0.05 was considered to be significant. Two‐way repeated measures ANOVA were used for the analysis of the papillary muscle and myocardial infarction models. One‐way repeated measures ANOVA was used for the rabbit haemodynamic study. The Sidak post hoc test was run only if the *F*‐value was statistically significant, and there was no significant variance in homogeneity. Data are presented as mean ± SEM. Prism 8.0.2 (GraphPad Software Inc., San Diego, CA, USA; RRID:SCR_002798) was used for statistical analysis.

### 2.16 Materials

#### 2.16.1 Study substance

ORM‐11372 was synthetized by Orion Pharma. To perform in vitro experiments, ORM‐11372 was dissolved in DMSO, to obtain a 10‐mM stock solution. Spiking solutions were prepared daily by diluting the stock solution with DMSO and used to prepare the final drug solutions with certain concentrations by diluting them with extracellular solutions (the vehicle concentration was either 0.1% or 0.3%). The solubility of ORM‐11372 in saline and 5% glucose solutions used for in vivo dosing was 5.3 and 5.4 mg·ml^−1^ respectively. ORM‐11372 was soluble in 10–100 μg·ml^−1^ of phosphate buffer (at pH 7.4). ORM‐11372 has the following drug like properties: The number of hydrogen bond acceptors is 1 (<10) and donors is 3 (<5), MW of free base is 282 (<500), and the predicted logP value is less than 5 (ADMET Predictor: 3.95, MOE: 3.413, and QikProp: 3.974).

#### 2.16.2 Other chemicals

The suppliers of other materials used are indicated as follows: Isoflurane (Forane®, Baxter), atipamezole (Antisedan, Orion Pharma), medetomidine (Domitor®, Orion Pharma), ketamine (Ketalar, Pfizer Oy Animal Health), lipofectamine (Invitrogen, USA), hygromycin B (Invitrogen), and buprenorphine (Temgesic®, INDIVIOR UK Ltd). All other chemicals and cell culture media were obtained from Sigma‐Aldrich.

#### 2.16.3 Cell lines and cell culture

NCX 1.1 was cloned, and plasmid was constructed by Orion Pharma Sf9 cells (ATCC), hiPSC‐derived CMs (Cor.4U CMs from Ncardia, Germany; Detachin™ Cell Detachment Solution, Genlantis), HEK 293 cells and IMR‐32 human neuroblastoma cells (ATCC), CHO cells hERG1a (KCNH2; Sophion Biosciences, Denmark), CHO cells human 5‐HT_2B_ receptors (Euroscreen, Belgium), and hSCN5A plasmid (OriGene Technologies Inc., Rockville, MD, USA).

### 2.17 Nomenclature of targets and ligands

Key protein targets and ligands in this article are hyperlinked to corresponding entries in the IUPHAR/BPS Guide to PHARMACOLOGY (http://www.guidetopharmacology.org) and are permanently archived in the Concise Guide to PHARMACOLOGY 2019/20 (Alexander, Christopoulos et al., 2019; Alexander, Fabbro et al., 2019; Alexander et al., 2019a, 2019b; Alexander, Mathie et al., 2019).

## 3 RESULTS

### 3.1 ORM‐11372 inhibits human and rat NCX activity

ORM‐11372 concentrations dependently inhibited the increase in intracellular calcium in the insect cell line expressing human NCX1.1. The insect cell line was used as the screening assay. The IC_50_ for the inhibition of NCX in the reverse mode was 6.2 ± 0.4 nM.

**FIGURE 3:**
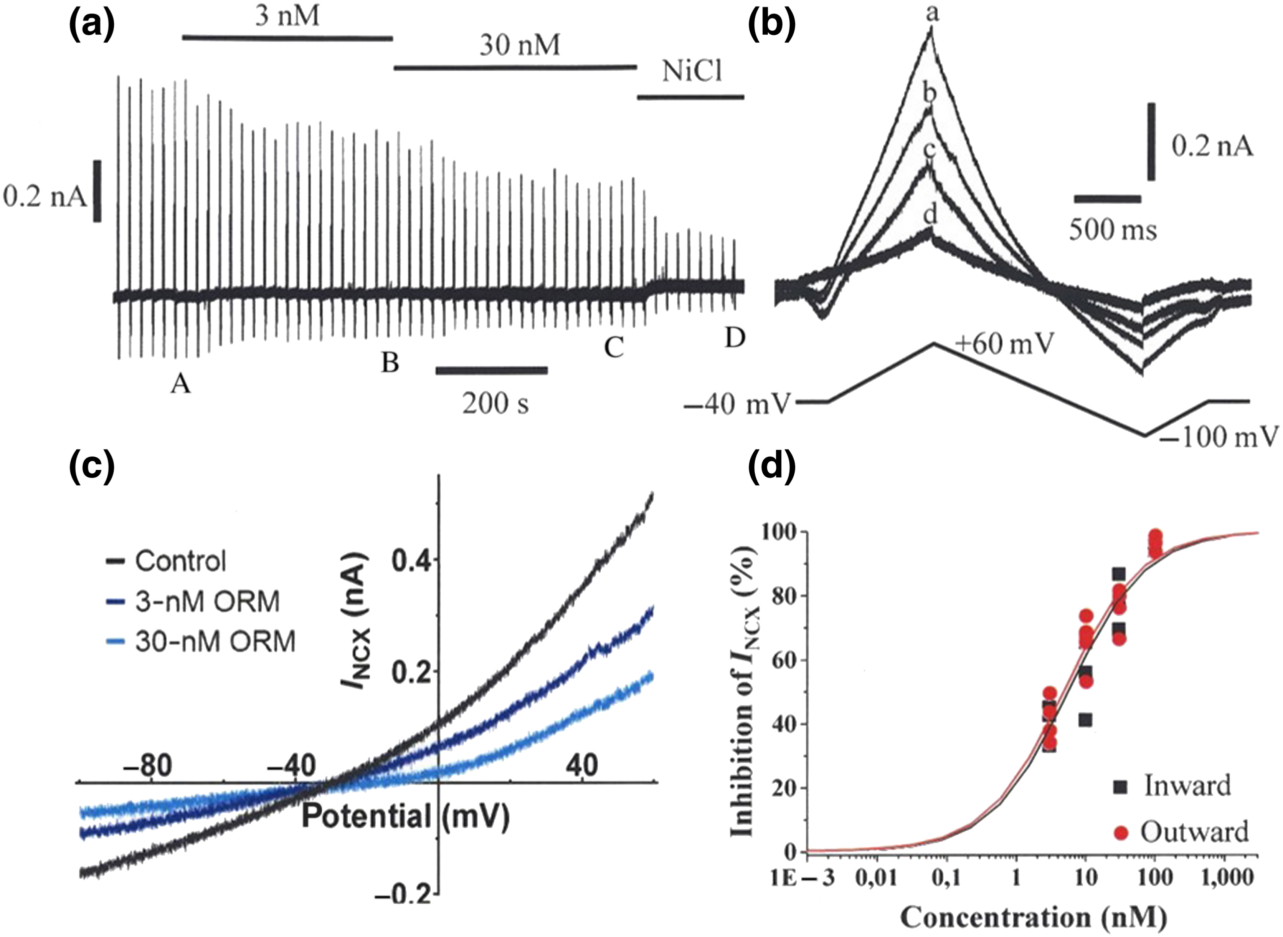
Effect of ORM‐11372 on the bidirectional *I*_NCX_ of human‐induced pluripotent stem cell (iPSc)‐derived cardiomyocytes. (a) The experimental time course included a ramp voltage protocol to be repeated every 20 s. The current traces at the labels (A: control; B and C: in the presence of 3‐ and 30‐nM ORM‐11372; and D: when *I*_NCX_ is fully blocked by 10‐mM NiCl_2_) are shown in (b) enlarged and superimposed (with the voltage protocol below) images. (c) The concentration–response curve for ORM‐11372 on the outward (3 nM, *n* = 4; 10 nM, *n* = 6; 30 nM, *n* = 4; and 100 nM, *n* = 3) and inward (3 nM, *n* = 4; 10 nM, *n* = 4; 30 nM, *n* = 3; and 100 nM, *n* = 2) *I*_NCX_ was determined at +60 and −100 mV respectively. The IC_50_ values are calculated using non‐linear regression. Data shown are individual values; n refers to the number of iPS derived cardiomyocytes

**FIGURE 4:**
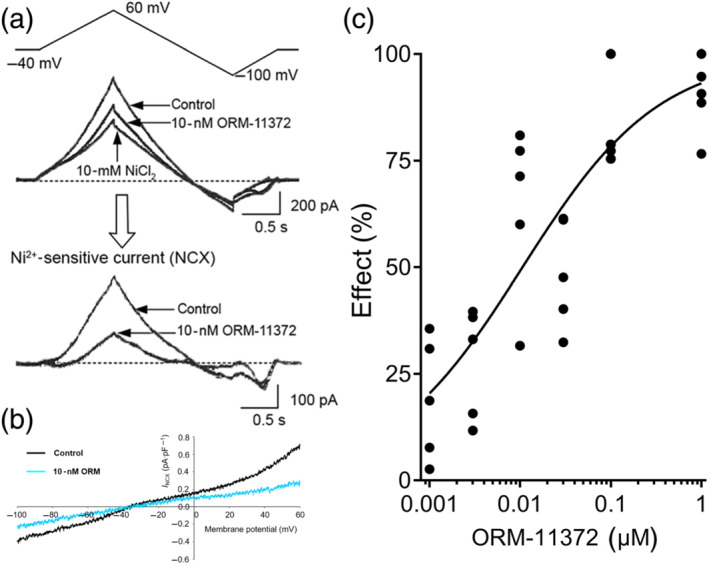
The effect of ORM‐11372 on the reverse sodium–calcium exchanger (NCX) current measured in rat ventricular cardiomyocytes is presented in panel (a). The top left panel (a) shows the voltage protocol applied during experiments: The *I*–*V* (current–voltage) relationship of the Na^+^/Ca^2+^ exchanger current was measured through the use of ramp pulses at 20‐s intervals. The ramp pulse initially lead to depolarization from the holding potential of −40 to 60 mV, at a rate of 100 mV·s^−1^, followed by hyperpolarization to −100 mV, and depolarization back to the holding potential. The middle panel illustrates original current records in the absence (control) and presence of 10‐nM ORM‐11372 and after applying 10‐mM NiCl_2_. The Ni^2+^‐sensitive current traces clearly show that 10‐nM ORM‐11372 effectively inhibits the reverse NCX current. Example of the *I*–*V* (current–voltage) relationship of the Na^+^/Ca^2+^ exchanger current is shown in panel (b). The magnitude of reverse NCX current measured at 20 mV was reduced by ORM‐11372 in a concentration‐dependent manner in rat ventricular cardiomyocytes in panel (c). The concentration–response curve for ORM‐11372 on the outward (*n* = 5) and inward (*n* = 3) *I*_NCX_ was determined at +60 and −100 mV respectively. The IC_50_ value for the outward *I*_NCX_ current is calculated using non‐linear regression. Data shown are individual values; *n* refers to the number of rat primary ventricular cardiomyocytes

The effects of ORM‐11372 for NCX was subsequently studied using the whole‐cell patch‐clamp technique in human iPS‐derived CMs (hiPSC‐CMs; Figure 3) and rat ventricular CMs (Figure 4). The solutions and experimental protocol used to measure the bidirectional NCX current (*I*_NCX_) were identical for both preparations. As shown in Figure 3a, the current was recorded in response to repetitive voltage ramp pulses first in a K^+^‐free bath solution after blocking Na^+^, Ca^2+^, K^+^, and Na^+^/K^+^ pump currents, to yield the baseline, and during the addition of ORM‐11372. Finally, when the highest concentration of ORM‐11372 had reached a steady state, 10‐mM NiCl_2_ was added to completely block *I*_NCX_. Examples of individual trace currents under different conditions are shown for a hiPSC‐CM in Figure 3b and for a rat CM in Figure 4a. ORM‐11372 decreased both the outward and inward currents. The IC_50_ values for the outward and inward *I*_NCX_ (i.e., the Ni^2+^‐sensitive current) of hiPSC‐CMs were 4.8 and 5.6 nM respectively (Figure 3c). In rat primary ventricular CMs, the magnitude of outward *I*_NCX_ (the reverse mode) with an IC_50_ of 11.3 nM (Figure 4b) was reduced by ORM‐11372, in a concentration‐dependent manner. It was difficult to record inward *I*_NCX_ (the forward mode) from rat CMs. We therefore altered the experimental conditions to enhance its magnitude (see Section 2). At 10 nM, the approximate IC_50_ on the outward *I*_NCX_, ORM‐11372 inhibited inward *I*_NCX_ by 53.8 ± 3.9%.

### 3.2 Comparison with known NCX inhibitors

**FIGURE 5:**
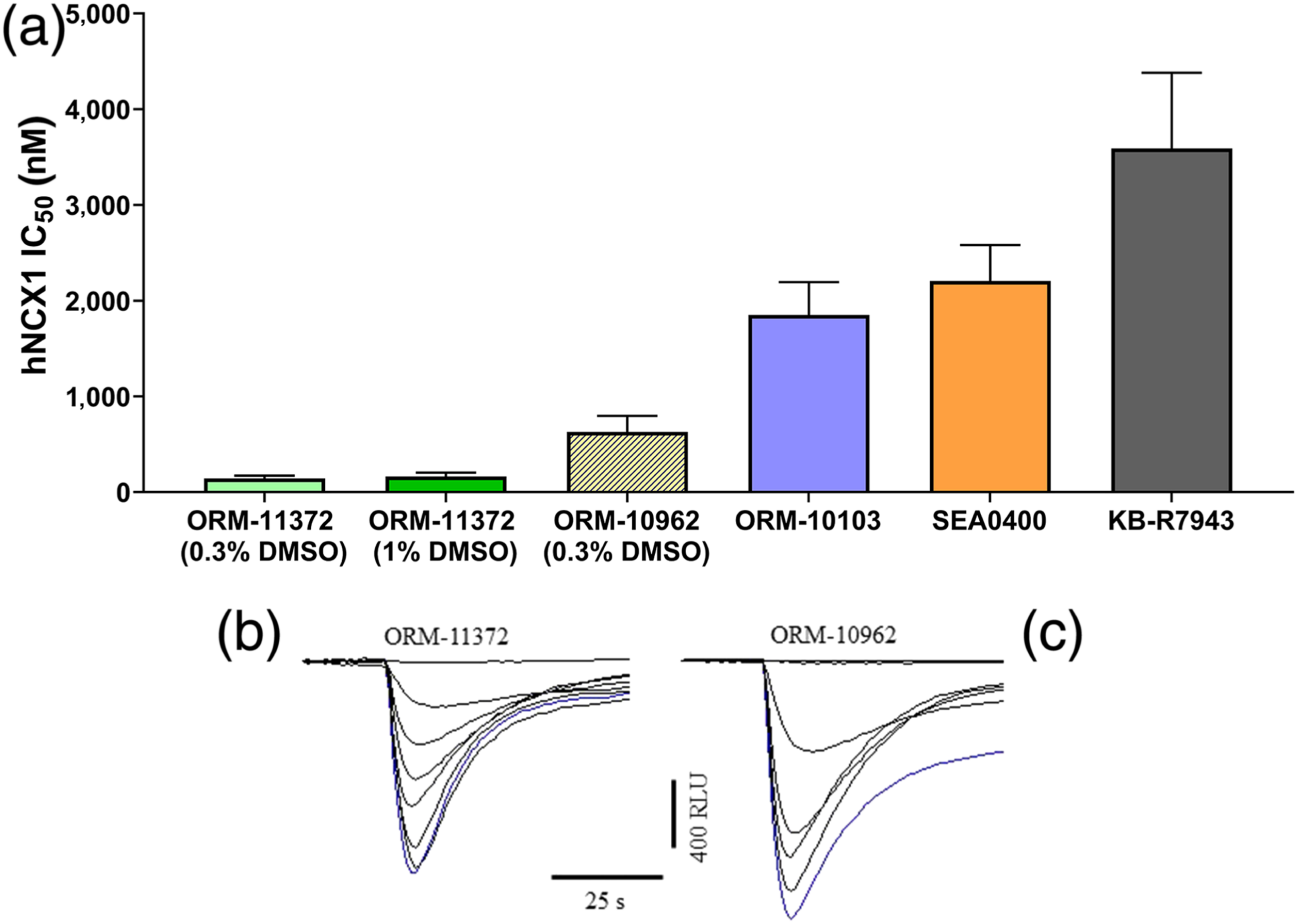
The effects of selected NCX inhibitors were tested on the forward mode in confirmatory hNCX1 inhibition assays in HEK293 cells. In (a), data shown are means ± SEM from *n*=5 assays. In (b), experimental records show the effects of increasing concentrations of ORM‐11372 (0.001, 0.003, 0.01, 0.03, 0.1, 0.3 and 1μM) and in (c), of ORM‐10962 (0.03, 0.1, 0.3, 1, 3 and 10μM) to decrease Ca^2+^ efflux (downward deflection) from control conditions (blue trace). Each assay derived from one plate with four replicates per plate

Further confirmation of the inhibitory effects of ORM‐11372 was sought in the hNCX1 fluorescence calcium flux assay available at Charles River. In experimental conditions increasing intracellular calcium (by the presence of thapsigargin and FCCP) and eliciting NCX forward mode activity (switching from Na^+^‐free to Na^+^‐containing buffer), ORM‐11372 concentration‐dependently inhibited the calcium efflux signal (Figure 5a) with an IC_50_ of 142 and 164 nM (0.3% and 1% DMSO, respectively; NS). The hNCX1 antagonist of five other compounds was also assessed in this assay. ORM‐11372 was the most potent inhibitor (Figure 5b) followed by ORM‐10962 (Figure 5c), ORM‐10103, SEA0400, KB‐R7943, and lastly SN‐6 whose IC_50_ appears >100 μM.

### 3.3 Selectivity

**FIGURE 6:**
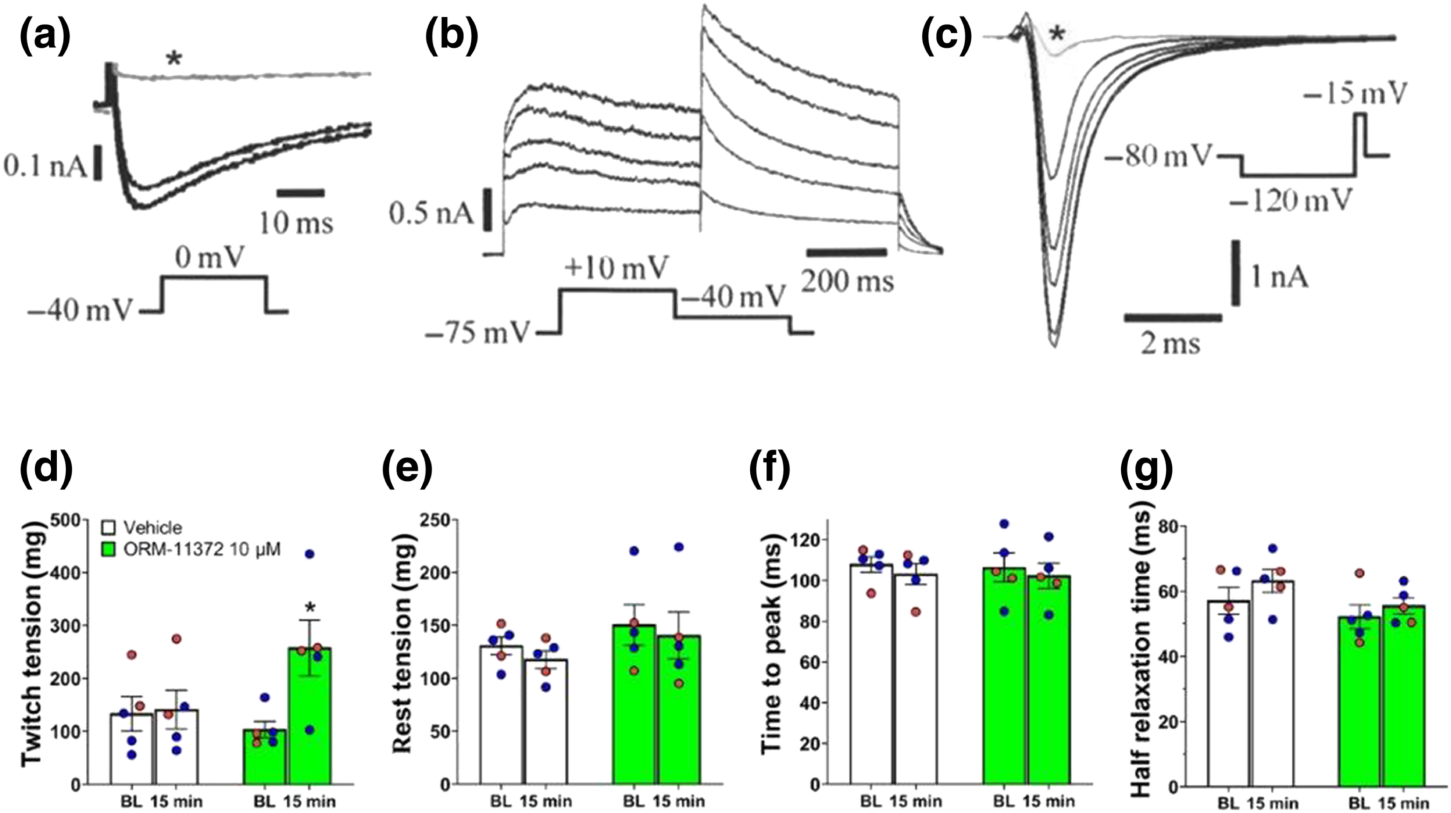
Effects of ORM‐11372 on various cardiac ion channel currents of induced pluripotent stem cell (iPSc)‐derived cardiomyocytes (illustrative figures in panels a–c) and contraction force in guinea pig papillary muscle (panels d–g). (a) The L‐type *I*_Ca_ was minimally inhibited by 1‐μM ORM‐11372. (b) The concentration‐dependent inhibition of K_V_11.1 current by 0.3‐, 3‐, 10‐, and 30‐μM ORM‐11372 and (c) Na_V_1.5 current by 1‐, 3‐, 10‐, and 30‐μM ORM‐11372. The applied voltage protocols are shown as insets. The traces marked by asterisk in (a) and (c) are the currents in the presence of 100‐nM nitrendipine and 2‐mM lidocaine respectively. (d) ORM‐11372 increased twitch tension, which indicates increased SR load. (e) ORM‐11372 did not increase resting tension, that is, no increase in diastolic calcium. (f) ORM‐11372 did not affect time to peak demonstrating that calcium release from ryanodine receptors is normal. (g) Also, half relaxation time was unchanged showing that SERCA function remains normal. Data shown are means ±SEM (*n* = 5) and individual values with red and blue circles for female and male respectively. Two‐way ANOVA followed by Sidak's multiple comparison test. *P < 0.05, signficant effect of ORM‐11372; two‐way ANOVA followed by Sidak's multiple comparison test. Pacing rate 1 Hz. Temperature 37°C

The functional selectivity of ORM‐11372 towards the L‐type Ca^2+^ channel was first tested with the IMR‐32 neuroblastoma cell line, using fluorometric images of intracellular changes in calcium levels. Depolarization of undifferentiated IMR‐32 cells by KCl addition induced an increase in intracellular calcium levels, which can be suppressed by pre‐incubation with verapamil or the 1,4‐dihydropyridine nicardipine (Sher et al., 1988). L‐type calcium channels mediated changes in intracellular calcium levels, and these were concentration‐dependently inhibited by ORM‐11372 with an IC_50_ of 6.1 μM. Further electrophysiological studies were then performed with hiPSC‐CMs and rat ventricular CMs. The inward *I*_CaL_ was reduced by 1‐μM ORM‐11372 at 0 mV by 14.1% in hiPSC‐CMs (Figure 6a), and a 7.8% inhibition was observed in rat CMs (Figure S16A,B; individual inhibition values at 1 and 10 μM are shown in Tables S3 and S4, respectively). However, when rat ventricular CMs were exposed to ORM‐11372 at a concentration of 10 μM, *I*_CaL_ was significantly decreased (Figure S16C). *I*_CaL_ inhibition exhibited voltage dependency, which was greater (59–69% vs. 36–39%) at more negative activating test potentials (−35 to −20 vs. 0 to 40 mV).

The effect of ORM‐11372 on other cardiac ion channel currents was investigated. The reduction in the human alpha subunit of a potassium ion channel (K_V_11.1) current (*I*_hERG_) was initially examined using stably expressing cells on an automated patch‐clamp instrument and then with the manual patch‐clamp. ORM‐11372 concentration‐dependently inhibited *I*_hERG_ (Figure 6b); IC_50_ values were 19.2 and 10.0 μM, using the automated and manual patch‐clamp methods respectively (Figure S15A). ORM‐11372 significantly altered the voltage dependency of the *I*_hERG_ activation curve (Figure S15C) and displayed significant voltage‐dependent blockage. While *I*_hERG_ inhibition was minimal at −30 and −20 mV, inhibition increased markedly at −10 and 0 mV and reached a steady state between 10 and 60 mV, that is, at potentials where the channels were fully activated (Figure S15D). Additionally, ORM‐11372 concentration‐dependently inhibited the human voltage‐gated sodium channel, alpha subunit 5 (Na_V_1.5) current (Figure 6c), with an IC_50_ value of 23.2 μM.

ORM‐11372 increased twitch tension compared with values in the vehicle group (Figure 6d), but did not increase rest tension (−8 ± 2% vs. −10 ± 1% in vehicle, Figure 6e) and did not affect time to peak (−4 ± 1% vs. −5 ± 2% in vehicle, Figure 6f) or the half relaxation time (Figure 6g).

The selectivity profile of ORM‐11372 was additionally evaluated in binding assays for a diverse panel of targets. While minor effects such as those for the Na^+^/K^+^‐ATPase or ryanodine receptor (11% and 15% inhibition) were seen, with a screening concentration of 10 μM, specific ligand‐binding inhibition was significant (i.e., >50%) at peripheral benzodiazepine receptor, 5‐HT_2A_ and 5‐HT_2B_ receptors, and at the noradrenaline transporter. The latter three targets were studied functionally. The uptake of noradrenaline into rat hypothalamus synaptosomes was inhibited by ORM‐11372 in a concentration‐dependent manner; the IC_50_ value was 1.7 μM. Neither agonism nor antagonism was seen with ORM‐11372 (maximum concentration: 10 μM) at 5‐HT_2A_ receptors. Similarly, no agonist activity was evident at 5‐HT_2B_ receptors but 10‐μM ORM‐11372 did moderately inhibited (38%) the 5‐HT_2B_ receptor‐mediated increase in intracellular calcium evoked by 10‐nM 5‐HT.

### 3.4 Human ventricular trabeculae

**FIGURE 7:**
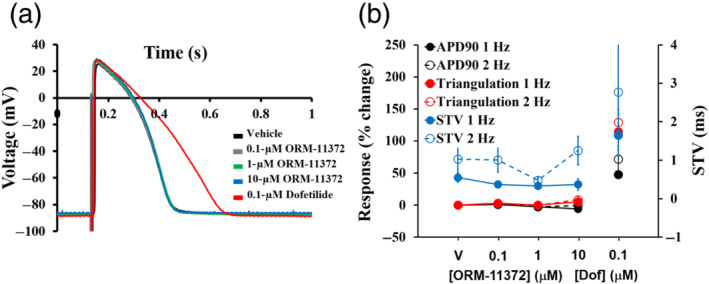
Effects of ORM‐11372 on action potentials (APs) in human ventricular trabeculae (*n* = 2, two replicates). (a) Typical APs recorded from a human ventricular trabecula at a pacing rate of 1 Hz in the presence of vehicle (V) control and after exposure to ORM‐11372 (0.1, 1, and 10 μM) and 0.1‐μM dofetilide (Dof) (the positive control). (b) Mean changes in APD_90_, triangulation, and short‐term variability analysis of AP duration (STV) values were in cadence when trabeculae were incubated with ORM‐11372 and Dof at 1 and 2 Hz. Notably, the effects of ORM‐11372 and Dof on APD_90_/triangulation activity and STV are plotted on a separate Y‐axis. Data shown are individual values; *n* refers to the number of human hearts, and replicates refer to the number of trabeculae in the same heart

ORM‐11372 caused no significant changes in APD_30_, APD_50_, and APD_90_ values up to a concentration of 10 μM, at both 1‐ and 2‐Hz pacing frequencies. However, there was a trend in the reduction in APD, particularly APD_30_, at 1 Hz (Figure S17); this seemingly contrasts with the significant shortening of hiPSC‐CM APD by ORM‐11372 (at 0.1 or 0.3 μM; Figure S18). As expected, the effect of the positive control (0.1‐μM dofetilide) was observed in trabeculae tested with ORM‐11372 (Figure 7); individual values are shown in Figure S17 (Page et al., 2016; Qu et al., 2017). Moreover, ORM‐11372 showed no effects on STV and triangulation, two pro‐arrhythmia markers that are used to identify pro‐arrhythmic risk (Page et al., 2016; Qu et al., 2017), at a pacing rate of 1 or 2 Hz, at any tested concentration (Individual values in Figure S19). However, the addition of 0.1‐μM dofetilide to the trabeculae tested with ORM‐11372 increased STV and triangulation (Figure S18). Taken together, these data clearly suggest that ORM‐11372 can be classified as devoid of pro‐arrhythmic risk.

### 3.5 In vivo haemodynamics in rat MI model

Infarcted areas were 14.5 ± 1.8% and 1.1 ± 0.6% in the MI and sham‐operated groups respectively (Figure S20). Percentage changes are calculated from absolute values. Infarctions caused the baseline of positive inotropic effect and relaxation to be reduced by 38% and 23% respectively. Heart rates (−3%) were unaffected, but systemic arterial pressure reduced by 20%.

**FIGURE 8:**
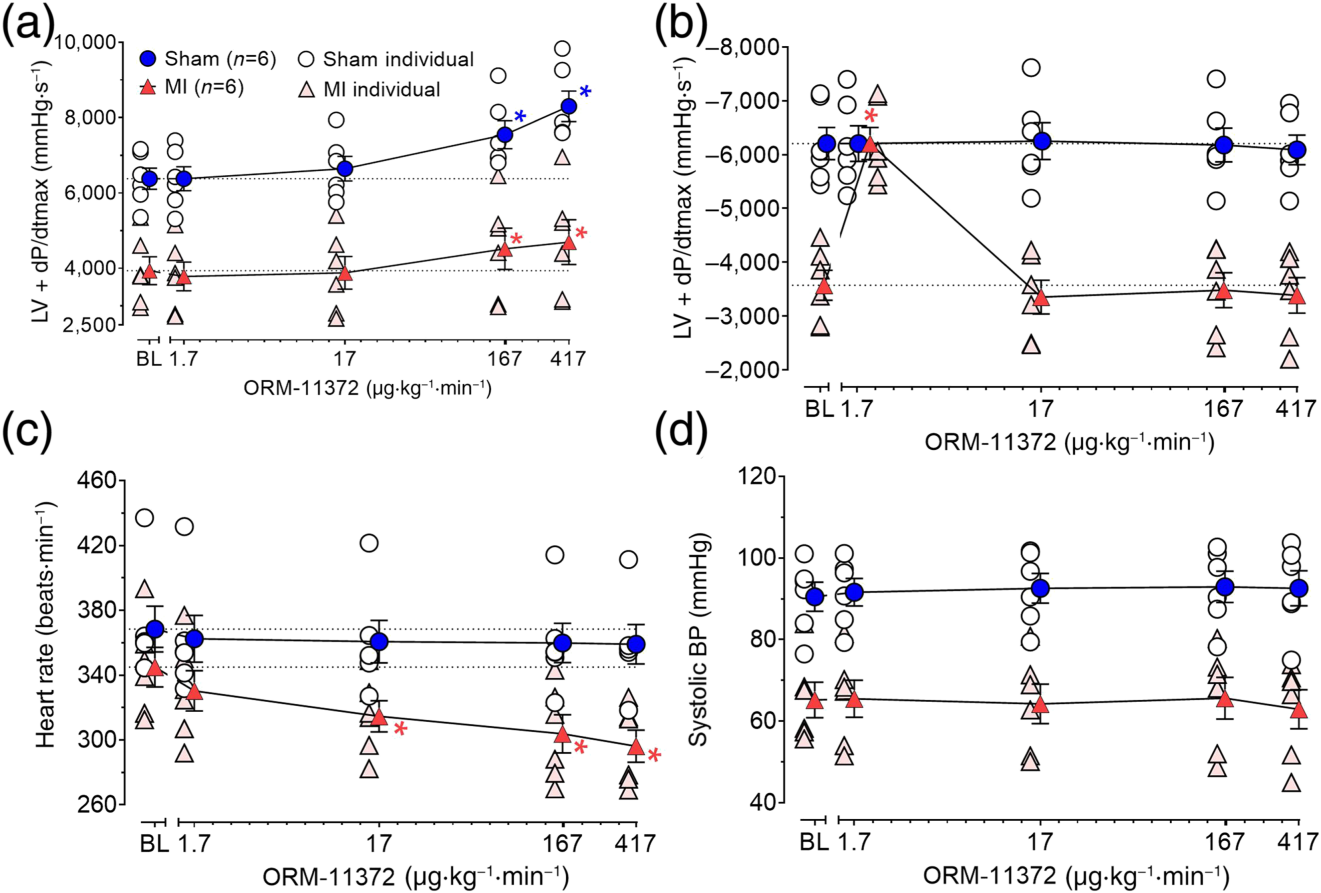
Effects of ORM‐11372 on haemodynamics in isoflurane‐anaesthetized rats, 7 days after the induction of myocardial infarction (MI, *n* = 6) or in sham rats (*n* = 6). Effects on the left ventricular contractility (LV + *dP*/*dt*_max_) are shown in panel (a), along with values for (b) relaxation (LV − *dP*/*dt*_max_), (c) heart rate, and (d) systolic arterial BP. Data shown are individual values with means ± SEM; n refers to number of rats. *P<0.05, significantly different from base line (BL); two‐way repeated measures ANOVA followed by Dunnett's post hoc test

In MI rats, ORM‐11372 improved maximally positive inotropic effect by 18 ± 6% and by 31 ± 6% in sham rats (Figure 8a). Maximal effects were observed at the highest infusion dose of 417 μg·kg^−1^·min^−1^, corresponding to free plasma concentrations of 6.7 ± 2.5 and 4.9 ± 0.4 nM. At the lowest infusion dose of 1.7 μg·kg^−1^·min^−1^, ORM‐11372 improved relaxation levels in the MI group by 24 ± 2% (Figure 8b). ORM‐11372 maximally reduced the HR in the MI group but not in the sham rats (Figure 8c). ORM‐11372 had no effect on the systemic arterial BP, in the MI or sham groups (Figure 8d).

### 3.6 In vivo haemodynamics in rabbits

**FIGURE 9:**
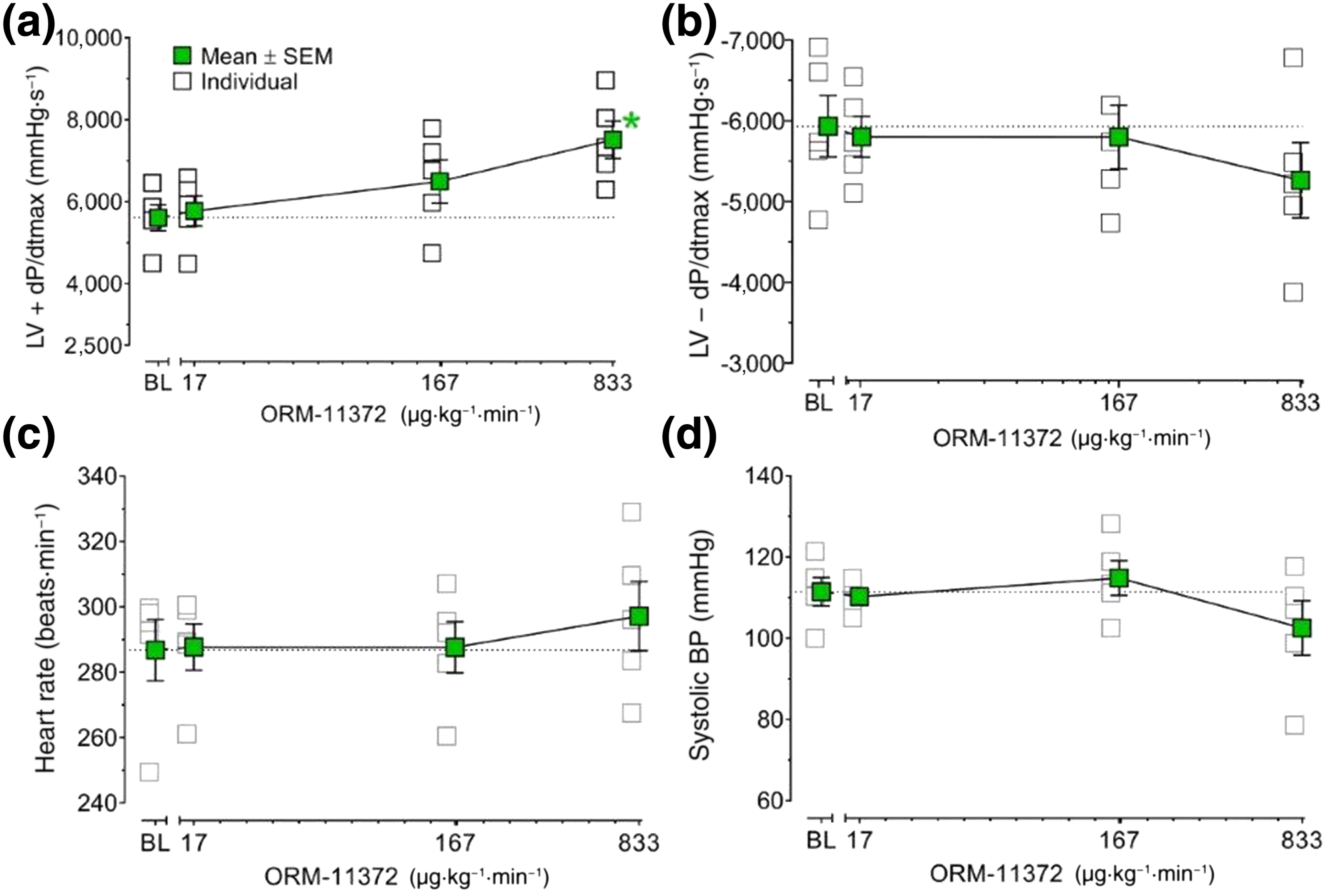
Effects of ORM‐11372 on haemodynamics in S‐ketamine‐anaesthetized rabbits (*n* = 5). The effects on (a) left ventricular contractility (LV + *dP*/*dt*_max_), (b) left ventricular relaxation (LV − *dP*/*dt*_max_), (c) heart rate, and (d) systolic arterial BP are shown. Data shown are individual values with means ± SEM; n refers to the number of rabbits. *P<0.05, significantly different from base line (BL); one‐way repeated measures ANOVA followed by Dunnett's post hoc test

ORM‐11372 induced a 35 ± 8% (calculated from absolute values) statistically significant increase in cardiac contractility in healthy rabbits, at an infusion rate of 833 μg·kg^−1^·min^−1^ (Figure 9a). At rates of 17–833 μg·kg^−1^·h^−1^, ORM‐11372 infusions did not have any effects on cardiac relaxation (Figure 9b), HR (Figure 9c), or systolic BP (Figure 9d) in healthy rabbits.

## 4 DISCUSSION

Although the first NCX inhibitor, exchanger‐inhibiting peptide (XIP), was a peptide, it enabled us to study the role of NCX under physiological and pathological conditions in cells and isolated tissues (Hobai et al., 1997). However, it took 20–30 min for NCX to be inhibited; it also inhibited SERCA and PMCA (Enyedi & Penniston, 1993). The next important step was the isolation and characterization of the naturally occurring NCX inhibitor (intrinsic factor‐inhibiting NCX [NCX_IF_]) (Hiller, Shpak, Shavit, Shpak, & Khananshvili, 2000). It was the first specific, cell membrane‐penetrating NCX inhibitor (Shpak, Hiller, Shpak, & Khananshvili, 2003), but the structure of NCX_IF_ was not published.

The first generation compounds (Table S1) that were specifically targeted to inhibit NCX were used as pharmacological tools to assess the inotropic mechanisms of various drugs like cardiac glycosides (Ruch, Nishio, & Wasserstrom, 2003; Tanaka et al., 2007) and insulin (Hsu et al., 2006). The potency of the first generation NCX inhibitors was modest, and selectivity between NCX and other membrane currents was poor, especially towards the L‐type Ca^2+^ current (Birinyi et al., 2005). SEA0400 was positively inotropic in rats, but not in rabbits (Farkas et al., 2008), in which the role of NCX is similar to that of humans. For the second‐generation NCX inhibitor ORM‐10103 (Koskelainen et al., 2003), the selectivity between NCX and other currents was improved (Kormos et al., 2014). The following molecule ORM‐10962 (Otsomaa et al., 2004) exhibits even better potency and more improved solubility (Kohajda et al., 2016). Discovery of ORM‐11372 created the ground for the third‐generation NCX inhibitors with a unique structure combined with improved drug‐likeness.

In this paper, we show that ORM‐11372 inhibited NCX 1.1 reverse and forward mode currents with a similar potency in both human iPS and rat CMs. Furthermore, the inhibition of human and rat NCX 1.1 currents was shown to be of the same magnitude (IC_50_ was 5 vs. 10 nM). NCX 1.1 expression has been demonstrated in iPS‐CMs (Fine et al., 2013; Kodama et al., 2019). To our knowledge, the presence or absence of the other NCX subtypes in hiPS has not yet been reported. Furthermore, NCX 1.1 expression is tissue specific (Lee, Yu, & Lytton, 1994); it can be stated that ORM‐11372 is a potent and selective NCX 1.1 inhibitor compared with other cardiac ion channels, but NCX subtype selectivity is unknown. NCX has specialized role in ECC. Cell‐specific regulation of NCX1 expression is due to a cardiac specific promoter, which leads to very high expression of NCX 1.1 in cardiac tissue (Nicholas et al., 1998). Hence, the effect of ORM‐11372 is cardiac specific. In earlier studies, it was speculated that positive inotropic effects could be attributable to the asymmetrical blocking of NCX (Oravecz et al., 2018). ORM‐11372 induced positive inotropy in healthy and MI rats, although it symmetrically inhibited both inward and reverse NCX currents. In rat plasma, the effective free concentration range of ORM‐11372 was from 4 to 300 nM (Figure S20), corresponding to the NCX inhibition range of IC_25_ to IC_80_ in rat CMs (Figure 4c). ORM‐11372 induced positive inotropy even in vivo, in the healthy rabbits, in contrast to SEA0400 (Farkas et al., 2008). The fact that the ORM‐11372 is more than 10 times more potent than SEA0400, as a NCX inhibitor, explains its positive inotropic effect in rabbits. This was also confirmed in hNCX1–HEK assay (Figure 5). ORM‐11372 increased twitch tension (Figure 6d), which indicates increased SR load. Furthermore, unlike SEA0400, ORM‐11372 inhibited NCX 1.1 current selectively, and other CM currents were negligibly inhibited.

ORM‐11372 had no effect on relaxation levels in normal rats and rabbits, but at the lowest dose, it improved relaxation levels in MI rats. Rat haemodynamics is subject to limitation of the fact that rat CM relaxation depends less on NCX and more on the other Ca^2+^ handling mechanisms. An earlier study conducted in vitro using SEA0400 showed that NCX inhibition caused deterioration in transient Ca^2+^ levels, but not mechanical relaxation (Szentandrassy et al., 2008). An ex vivo study using rat isolated perfused hearts with artificial ionic modification, demonstrated that NCX inhibition also depressed mechanical relaxation levels (Chen et al., 2012). However, it must be emphasized that the earlier studies were performed using the weaker and less selective inhibitor SEA0400 (Table 1), under artificial conditions. ORM‐11372 did not affect relaxation in guinea pig papillary muscle (Figure 6g) or in rabbits in vivo*,* because of the activation of compensatory Ca^2+^ handling mechanisms in CMs, as increased intracellular Ca^2+^ levels enhance SERCA and/or PMCA activity. Furthermore, ORM‐11372 had no effects on resting tension (Figure 6e) indicating no increase in diastolic calcium. ORM‐11372 did not either affect time to peak (Figure 6f), which demonstrated that calcium release from ryanodine receptors is normal. Our findings are in line with the study using NCX_IF_. Haemodynamic assessments in the present study were made only in male rats and rabbits, which might introduce potential bias due to sex differences. Our results with human trabeculae and guinea pig papillary muscle did not show any differences between sexes, although the number of experiments are low. However, calcium handling activity and NCX expression level in CMs have been shown to vary with sex, age and regional expression of NCX (Janczewski & Lakatta, 2010). Therefore, the role of NCX in calcium handling between sexes should be systematically further explored.

ORM‐11372 selectively inhibited *I*_NCX_ over the K_v_11.1 current and provided a safety margin (ratio ≥2,000) of well beyond 30‐fold, which was considered adequate for ensuring an acceptable degree of safety from arrhythmogenesis (Redfern et al., 2003). This selectivity was also confirmed in human ventricular tissue. Thus, ORM‐11372 can be classified as being non‐pro‐arrhythmogenic in humans.

Although the NCX current seems to be crucial for beat generation (Groenke et al., 2013), even a small NCX current is enough to maintain the heart beat (Gao et al., 2013). In the present study, ORM‐11372 had no effects on HR in vivo in rats or rabbits, and up to 80% NCX can be inhibited without any effects on the HR (Figure S20). In vascular arterial cells, NCX contributes to the maintenance of the myogenic tone (Zhang et al., 2010). In a manner similar to SEA0400 (Yatabe et al., 2015), ORM‐11372 had no effects on systolic BP. These pharmacological properties resulted in unique positive inotropic effects without the risk of hypotension, increase in HR, or impairment of left ventricular relaxation.

During the discovery of ORM‐11372, multiple iterative DMTA cycles (Design, Make, Test, Analyse) of optimization (Andersson et al., 2009) were performed in compliance with the 3R principles, using the protein‐ and cell‐based in vitro methods, before selecting of the compounds for in vivo studies. Group size was optimized but was large enough to obtain reliable results. Animals were also allowed to become habituated to the procedures, and the necessary analgesia was used. Rats and rabbits were selected to ensure similar responses and improved prediction capabilities from in vitro to in vivo studies, which ultimately would be translated into better efficacy and safety in human studies. To improve pharmacokinetic and pharmacodynamic analyses, the plasma levels of ORM‐11372 were monitored at the end of each pharmacodynamic experiment.

A critical feature of the ORM‐11372 scaffold was the bridged aniline structure with hydrogen bond formation between the A‐ and B‐ring systems (Figures 1 and 2). The first‐ and the second‐generation NCX inhibitors lack this hydrogen bond donor in their scaffold. Furthermore, the activity of the ORM‐11372 series exhibited better potency in the screening assay than the known NCX inhibitors (Tables S1 and S2). This indicates that an additional specific binding interaction was identified between the ligand and the NCX protein. However, the finding remains speculative due to the lack of other experimental evidence, for example, protein–ligand crystal structure. The crystal structure of the mammalian NCX and its splice variants are still unavailable, though the crystal structure of the NCX protein of archaebacterial *Methanococcus jannaschii* has been published in 2012 (Liao et al., 2012). There are known structural differences between the prokaryotic and eukaryotic NCX proteins that might lead to differences in the potential binding pockets and selectivity (John, Liao, Jiang, & Ottolia, 2013; Khananshvili, 2014). Thus, evidence regarding the binding pocket for ORM‐11372, as well as for the other NCX inhibitors is yet to be provided.

In conclusion, ORM‐11372 is the first in a class of the third generation of NCX 1.1 inhibitors with a unique scaffold and improved profile. ORM‐11372 is the most potent and selective NCX 1.1 inhibitor decribed so far. ORM‐11372 selectively inhibited cardiac NCX 1.1 currents and induced positive inotropic effects in healthy and MI rats, as well as in rabbits. ORM‐11372 exhibited a better haemodynamic profile and similar positive inotropic effects, as compared with dobutamine, but did not cause an increase in HR or decrease in systolic BP. Interestingly, at a low dose, ORM‐11372 improved relaxation in the MI model. Studies involving human tissues support the classification of ORM‐11372 as a non‐pro‐arrhythmic inhibitor of NCX. Thus, ORM‐11372 can be used as a research tool in further studies of the role and function of NCX1.1 in vitro and in vivo.

## AUTHOR CONTRIBUTIONS

L.O. had the main responsibility of devising and conceptualizing the research project. J.L., N.J., L.V., A.V., E.M., and J.Gy.P. were responsible for the pharmacological conceptualization. L.O., J.L., G.W., H.C., A.‐P.K., K.S., T.K., S.‐E.P., P.F., A.H., and Z.K. performed the research and analysed the data. N.A.‐G., A.G., P.E.M., and G.P. were responsible for designing the human tissue studies. L.O. and J.L. had the principal responsibility of writing and drafting the manuscript. N.A.G., E.M., and N.J. critically reviewed the original draft of the manuscript and contributed to the writing process. G.W. critically commented on the manuscript.

## CONFLICT OF INTEREST

L.O., J.L., G.W., H.C., A.‐P.K., K.S., T.K., S.‐E.P., P.F., and A.H. are or were employees of Orion and may own Orion Corporation stocks and shares. N.A.‐G., A.G., P.E.M., and G.P. are employees of AnaBios Corporation and may own AnaBios stocks and shares.

## DECLARATION OF TRANSPARENCY AND SCIENTIFIC RIGOUR

This Declaration acknowledges that this paper adheres to the principles for transparent reporting and scientific rigour of preclinical research as stated in the BJP guidelines for Design & Analysis and Animal Experimentation, and as recommended by funding agencies, publishers and other organisations engaged with supporting research.

## Supporting information


**Table S1.** Summary table of published NCX inhibitor chemical structures and originators
**Table S2.** Supporting Information
**Table S3.** Effect of 1 μM ORM‐11372 on L‐type calcium current amplitude at different test potentials in rat ventricular myocytes
**Table S4.** Effect of 10 μM ORM‐11372 on L‐type calcium current amplitude at different test potentials in rat ventricular myocytes
**Table S5.** Human donor heart demographics
**Figure S1.** Supporting Information
**Figure S2.** Supporting Information
**Figure S3.** ORM‐120407
**Figure S4.** ORM‐11023
**Figure S5.** ORM‐11024
**Figure S6.** ORM‐11165
**Figure S7.** ORM‐11190
**Figure S8.** ORM‐11217
**Figure S9.** intermediate of ORM‐11372
**Figure S10.** ORM‐11372
**Figure S11.** ORM‐11817
**Figure S12.** ORM‐11875
**Figure S13.** ORM‐11863
**Figure S14.** ORM‐11298
**Figure S15.** Supporting Information
**Figure S16.** Supporting Information
**Figure S17.** Supporting Information
**Figure S18.** Supporting Information
**Figure S19.** Supporting Information
**Figure S20.** Haemodynamics in healthy rats
Myocardial infarction model in rats.

Supporting information

## Data Availability

The data that support the findings of this study are available from the corresponding author upon reasonable request. Some data may not be made available because of privacy or ethical restrictions.
