# Supplementary material for: Discovery and characterization of ORM‐11372, a novel inhibitor of the sodium‐calcium exchanger with positive inotropic activity
Source: Br J Pharmacol. 2020 Nov 10;177(24):5534–54. doi: 10.1111/bph.15257 (PMC7707092; doi:10.1111/bph.15257)
Supplement: Supplementary file 1 — Table S1. Summary table of published NCX inhibitor chemical structures and originators Table S2. Supporting Information Table S3. Effect of 1 μM ORM‐11372 on L‐type calcium current amplitude at different test potentials in rat ventricular myocytes Table S4. Effect of 10 μM ORM‐11372 on L‐type calcium current amplitude at different test potentials in rat ventricular myocytes Table S5. Human donor heart demographics Figure S1. Supporting Information Figure S2. Supporting Information Figure S3. ORM‐120407 Figure S4. ORM‐11023 Figure S5. ORM‐11024 Figure S6. ORM‐11165 Figure S7. ORM‐11190 Figure S8. ORM‐11217 Figure S9. intermediate of ORM‐11372 Figure S10. ORM‐11372 Figure S11. ORM‐11817 Figure S12. ORM‐11875 Figure S13. ORM‐11863 Figure S14. ORM‐11298 Figure S15. Supporting Information Figure S16. Supporting Information Figure S17. Supporting Information Figure S18. Supporting Information Figure S19. Supporting Information Figure S20. Haemodynamics in healthy rats Myocardial infarction model in rats. [file BPH-177-5534-s001.pdf]

ONLINE SUPPLEMENTARY MATERIAL

Discovery and characterization of ORM-11372, a unique and positively inotropic sodium-calcium exchanger/inhibitor

Leena Otsomaa[1], Jouko Levijoki[1], Gerd Wohlfahrt[1], Hugh Chapman[1], Ari-Pekka Koivisto[1], Kaisa Syrjänen[1], Tuula Koskelainen[1], Saara-Elisa Peltokorpi[1], Piet Finckenberg[2], Aira Heikkilä[1], Najah Abi-Gerges[3], Andre Ghetti[3], Eero Mervaala[2], Norbert Nagy[4,5], Zsófia Kohajda[4], Norbert Jost[4,5,] László Virág[5], András Varró[4,5], Julius Gy. Papp[4,5]

[1]Orion Pharma R&D, Espoo, Finland, [2]Department of Pharmacology, Faculty of Medicine, Helsinki, Finland,[3]ANABIOS Corporation, 3030 Bunker Hill Street #312, San Diego, CA 92109,
[4]MTA-SZTE Research Group of Cardiovascular Pharmacology, Hungarian Academy of Sciences, Szeged, [5]Department of Pharmacology and Pharmacotherapy, Interdisciplinary Excellence Centre, Faculty of Medicine, University of Szeged, Szeged, Hungary, Hungary

Corresponding author: Leena Otsomaa, Orion Pharma R&D, Orionintie 1, P.O.BOX 65
FIN-02100 Espoo, Finland; e-mail: leena.otsomaa@orionpharma.com

Table S1 Summary table of published NCX inhibitor chemical structures and originators

Ref.	Code	Structure	Originator	Year
(Hori, Yamamoto, Ootaka, Nakajima, Harada & Morita, 1997)	KB-R7943	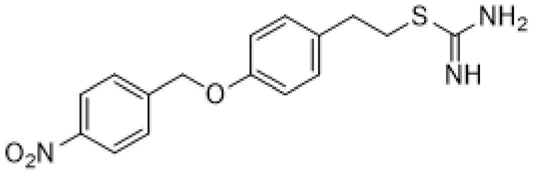	Kanebo 1st Generation	1997
(Iwamoto, Inoue, Ito, Sakaue, Kita & Katsuragi, 2004)	SN-6	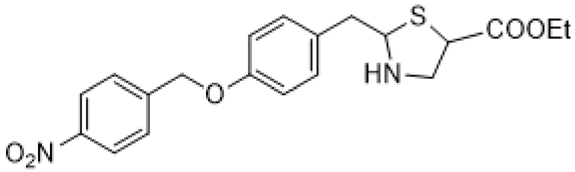	Fukuoka University 1st Generation	2002
(Ota, Nakanishi, Aibe, Taguchi & Tomisawa, 1999)	SEA 0400	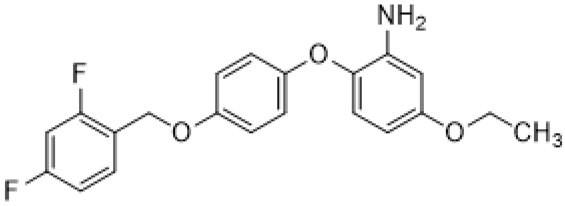	Taisho Pharma-ceuticals 1st Generation	1999
(Koskelainen et al., 2003)	ORM-10103	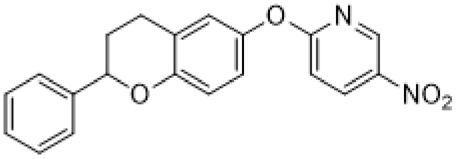	Orion 2nd Generation	2003
(Otsomaa, Koskelainen, Karjalainen, Rasku, Pollesello & Levijoki, 2004)	ORM-10962	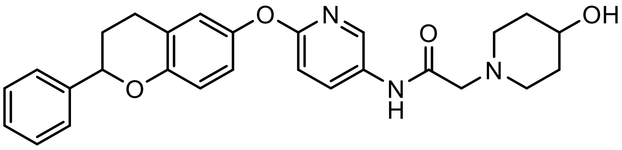	Orion 2nd Generation	2004
(Kakefuda et al., 2003; Kuramochi, Kakefuda, Yamada, Tsukamoto, Taguchi & Sakamoto, 2005)	YM-244796	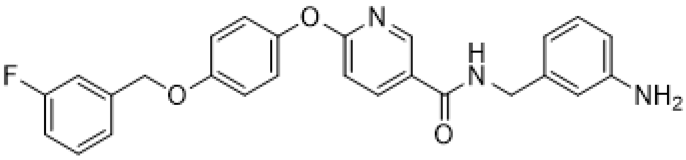	Astellas Pharma 1st Generation	2005
(Czechtizky et al., 2014)	-	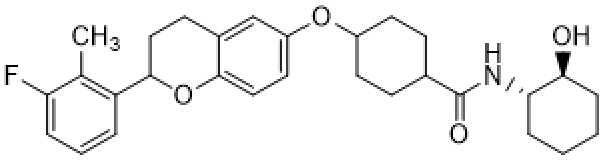	Sanofi 2nd Generation	2014
(Szakonyi & Fueloep, 2010)	GYKB 6635	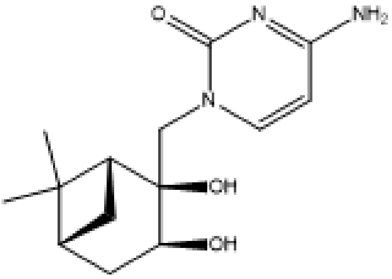	-	2010
(Otsomaa L., 2018)	ORM-11372	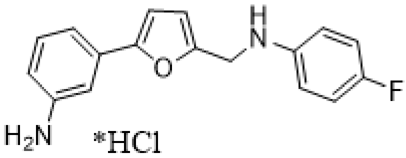	Orion 3rd Generation	2018

Table S2

Code	IC_{50} Sf9 nM	hNCX1-HEK nM	IC_{50} hiPS nM (reverse/ forward)	IC_{50} nM (reverse/ forward)	hERG μM	Type Ca^{2+} Channel μM	NCX inhibitor generation	MAO A/B inhibition % at 10 μM or IC_{50}	Solubility class
KB-R7943	3794	3590	-	Rat[1] 61/120 Rabbit[1] 309/243	0.089[2]	3.2[3]	1	-	Moderate
SN-6	16000	ND (>100000)	-	CCL39 fibroblast[4] 29000/ >30000	10[2]		1	38%/80%	Moderate
SEA 0400	376	2210	-		2.8	3.4	1	4340/176 nM	Insoluble
ORM-10103	176	1850	-	Dog 780/ 960	-	-	2	-	Insoluble
ORM-10962	197	630	-	Dog 55/ 67	3.4	11.4	2	11%/52%	Moderate
YM-244769	-	-	-	SH-SY5Y neuron[9] 96/68		>3[9]	1	-	
ORM-11372	6	142	5/6	Rat 11/ 10	10.6	6.1	3	29%/77%	Moderate

The solubility classes in pH 7.4 phosphate buffer: insoluble less than 10 μg/ml; moderate solubility between 10-100 μg/ml; soluble has better than 100 μg/ml solubility. ND denotes that IC_{50} could not be determined due to lack of effect.

Table S3. Effect of 1 µM ORM-11372 on L-type calcium current amplitude at different test potentials in rat ventricular myocytes

Control

Potential mV	L6125104 pA	Z6125105 pA	File_1559_003 pA	File_1559_001 pA	Z6126110 pA	MEAN pA	SEM pA	Cells
-35	-223.7	-156.1	-137.3	-77.3	-120.5	-143.0	24.0	5
-30	-471.4	-241.4	-208.6	-83.8	-230.3	-247.1	62.8	5
-25	-784.2	-383.5	-422.9	-97.6	-395.8	-416.8	109.2	5
-20	-880.0	-566.7	-957.7	-256.3	-637.1	-659.5	124.3	5
-15	-921.5	-926.4	-1346.5	-460.3	-1136.6	-958.3	147.1	5
-10	-1158.3	-1327.3	-1269.0	-541.6	-1555.3	-1170.3	170.0	5
-5	-1353.2	-1563.3	-1561.6	-525.5	-1776.3	-1356.0	218.1	5
0	-1509.7	-1646.5	-1475.7	-477.6	-1767.5	-1375.4	230.4	5
5	-1551.7	-1612.8	-1320.2	-434.3	-1680.5	-1319.9	229.6	5
10	-1498.8	-1493.5	-1130.9	-337.6	-1516.7	-1195.5	226.3	5
15	-1336.3	-1287.0	-924.6	-257.4	-1351.1	-1031.3	208.7	5
20	-1138.4	-1055.4	-692.7	-171.6	-1140.5	-839.7	186.3	5
25	-951.1	-816.7	-478.4	-119.8	-910.2	-655.3	157.6	5
30	-733.1	-592.9	-285.9	-116.8	-683.2	-482.4	119.9	5
35	-552.8	-391.0	-109.4	-42.6	-509.0	-321.0	104.0	5
40	-390.9	-209.9	40.4	-29.5	-332.3	-184.5	83.6	5
45	-263.7	-51.4	132.8	-68.3	-183.7	-86.9	67.3	5
50	-168.5	48.1	185.1	-7.6	-67.5	-2.1	59.0	5
55	-83.4	115.1	312.6	-3.7	26.7	73.5	67.7	5

1 µM ORM-11372

Potential mV	L6125106 pA	Z6125107 pA	File_1559_003 pA	File_1559_001 pA	Z6126112 pA	MEAN pA	SEM pA	Cells
-35	-197.4	-143.8	-151.4	-87.5	-106.9	-137.4	19.1	5
-30	-448.5	-202.2	-244.8	-128.7	-249.2	-254.7	53.1	5
-25	-669.6	-343.9	-565.8	-191.0	-371.0	-428.2	84.8	5
-20	-810.2	-523.0	-720.5	-412.8	-509.4	-595.2	73.4	5
-15	-992.6	-903.0	-1062.0	-501.6	-803.2	-852.5	97.9	5
-10	-1225.6	-1255.5	-1548.4	-525.7	-1196.7	-1150.4	168.4	5
-5	-1449.4	-1474.2	-1295.2	-524.7	-1397.7	-1228.2	178.5	5
0	-1620.3	-1530.4	-1248.6	-482.3	-1458.6	-1268.0	205.8	5
5	-1647.2	-1464.8	-1160.2	-477.1	-1416.0	-1233.1	204.4	5
10	-1542.2	-1334.2	-993.8	-349.8	-1309.9	-1106.0	208.4	5
15	-1380.1	-1150.7	-771.2	-280.8	-1129.8	-942.5	192.0	5
20	-1169.9	-945.6	-585.2	-200.6	-956.5	-771.6	170.9	5
25	-959.7	-734.0	-402.8	-122.6	-774.3	-598.7	149.1	5

30	-746.2	-551.4	-244.8	-58.4	-601.7	-440.5	125.7	5
35	-573.5	-373.9	-71.8	-5.4	-427.7	-290.5	108.4	5
40	-410.7	-231.0	56.8	43.0	-285.7	-165.5	92.7	5
45	-284.5	-108.8	111.4	66.1	-163.8	-75.9	73.3	5
50	-184.9	-19.5	243.4		-64.4	-6.3	90.3	4
55	-89.7	43.8	295.5		11.0	65.1	81.9	4

Table S4. Effect of 10 µM ORM-11372 on L-type calcium current amplitude at different test potentials in rat ventricular myocytes

Control

Potential mV	L6105103 pA	L6113103 pA	Z6111305 pA	Z6112210 pA	Z6112303 pA	Z6113110 pA	MEAN pA	SEM pA	Cells
-35	-78.9	-138.3	-207.5	-159.2	-160.5	-192.8	-156.2	18.5	6
-30	-159.5	-259.5	-582.8	-470.1	-317.3	-424.3	-368.9	62.6	6
-25	-248.7	-468.8	-1141.8	-914.1	-448.8	-783.5	-667.6	136.9	6
-20	-499.6	-619.4	-1503.9	-1269.5	-575.5	-1052.0	-920.0	169.9	6
-15	-965.5	-666.3	-1559.9	-1528.2	-790.2	-1255.3	-1127.6	154.5	6
-10	-1426.3	-756.1	-1736.2	-1998.9	-1142.7	-1626.0	-1447.7	181.7	6
-5	-1705.3	-998.4	-2008.5	-2369.3	-1494.0	-1997.6	-1762.2	195.2	6
0	-1796.7	-1260.3	-2079.5	-2403.9	-1733.3	-2098.6	-1895.4	160.5	6
5	-1751.2	-1379.8	-2026.6	-2281.9	-1776.7	-2033.6	-1875.0	127.0	6
10	-1616.0	-1407.0	-1879.1	-2070.0	-1748.8	-1910.5	-1771.9	96.2	6
15	-1425.4	-1312.3	-1683.8	-1817.9	-1594.4	-1692.7	-1587.7	76.5	6
20	-1230.1	-1161.7	-1457.7	-1543.5	-1302.0	-1451.2	-1357.7	60.8	6
25	-1009.5	-971.8	-1252.4	-1270.8	-1007.8	-1183.4	-1116.0	55.1	6
30	-817.6	-781.7	-1043.1	-1024.9	-805.5	-905.1	-896.3	46.8	6
35	-631.3	-606.7	-850.1	-808.7	-614.2	-682.1	-698.8	43.0	6
40	-462.5	-482.2	-653.5	-623.5	-469.2	-461.1	-525.3	36.1	6
45	-323.2	-258.1	-475.5	-472.2	-381.1	-337.1	-374.5	35.3	6
50	-236.3	-306.0	-333.4	-355.1	-323.8	-191.4	-291.0	25.9	6
55	-148.8	-153.8	-173.9	-250.6	-220.6	-2.7	-158.4	35.1	6

10 µM ORM-11372

Potential mV	L6105105 pA	L6113106 pA	Z6111307 pA	Z6112212 pA	Z6112305 pA	Z6113113 pA	MEAN pA	SEM pA	Cells
-35	-24.0	-71.0	-55.6	-46.0	-72.4	-115.8	-64.1	12.6	6
-30	-64.5	-115.4	-95.1	-99.4	-130.5	-190.7	-115.9	17.5	6
-25	-140.7	-173.3	-173.6	-181.5	-152.3	-283.0	-184.1	20.8	6
-20	-285.5	-251.0	-328.6	-356.8	-219.6	-455.0	-316.1	34.4	6
-15	-575.8	-371.3	-637.1	-633.4	-360.1	-575.4	-525.5	51.7	6
-10	-904.6	-608.1	-975.2	-964.9	-576.8	-814.6	-807.4	72.0	6
-5	-1199.2	-827.4	-1213.3	-1210.8	-762.1	-1045.8	-1043.1	83.1	6
0	-1320.4	-1010.2	-1301.7	-1262.2	-876.2	-1249.3	-1170.0	74.5	6
5	-1354.2	-1120.1	-1255.1	-1262.6	-895.7	-1299.3	-1197.8	68.2	6
10	-1250.7	-1082.5	-1149.2	-1138.1	-838.2	-1254.7	-1118.9	62.5	6
15	-1124.3	-994.1	-1015.8	-993.3	-783.3	-1108.3	-1003.2	49.8	6
20	-983.3	-883.4	-876.6	-843.6	-703.8	-952.4	-873.9	40.0	6
25	-808.8	-738.5	-701.0	-673.7	-606.3	-780.3	-718.1	30.2	6
30	-621.1	-582.6	-545.6	-536.9	-493.3	-589.2	-561.5	18.5	6
35	-478.0	-466.7	-403.6	-403.1	-398.0	-426.8	-429.4	14.3	6
40	-356.4	-330.6	-281.8	-321.8	-334.6	-300.3	-320.9	10.8	6
45	-248.3	-221.8	-168.5	-307.5	-267.4	-208.5	-237.0	19.8	6
50	-206.9	-228.7	-88.6	-238.7	-301.4	-70.2	-189.1	37.1	6
55	-136.7	-112.4	-18.3	-211.7	-193.4	-14.7	-114.5	34.3	6

Table S5. Human donor heart demographics

Heart #	Donor identifier	Age	Sex	Ethnicity	Body mass index	Cause of death	Ejection fraction*
1	160211HHA	57	F	Asian	23.4	CVA/Stroke	65%
2	160216HHA	57	M	Hispanic	20.3	Head trauma/Blunt injury	60%

*Ejection fraction was obtained from the donor echocardiography report.

Experimental of compound synthesis

Figure S1

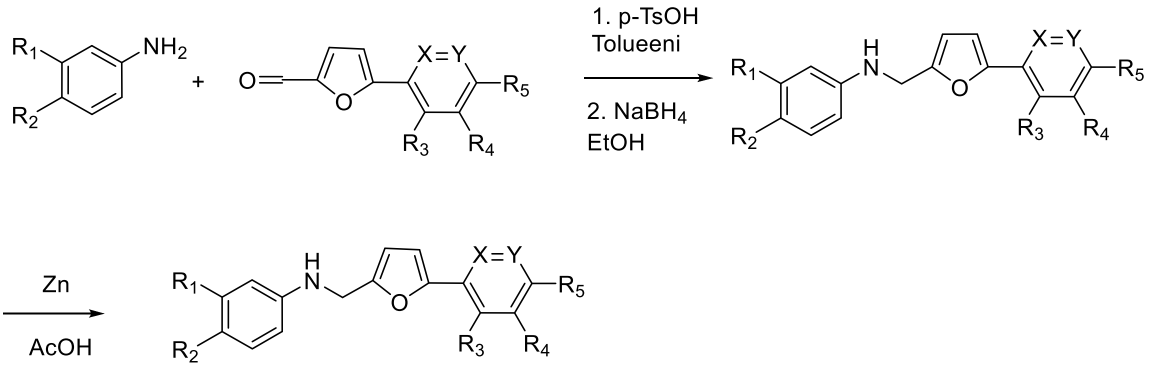

ORM-120407: R1 = Cl; R2 = F; R3 = NO2; R4, R5 = H; X, Y = C
ORM-11023: R1 = Cl; R2 = F; R3, R5 = H; R4 = NO2; X, Y = C
ORM-11024: R1, R2, R4, R5 = H; R3 = NO2; X, Y = C
ORM-11165: R1, R3 = Cl; R2 = F; R4, R5 = H; X, Y = C
ORM-11190: R1, R5 = Cl; R2 = F; R3, R4 = H; X, Y = C
ORM-11217: R1, R4 = Cl; R2 = F; R3 = H, R5 = H; X, Y = C
ORM-11372: R1, R3, R5 = H; R2 = F; R4 = NH2; X, Y = C
ORM-11817: R1, R2 = F; R3, R4, R5 = H; X = N; Y = C
ORM-11875: R1 = H; R2 = F; R3, R4, R5 = H; X = C; Y = N

Figure S2

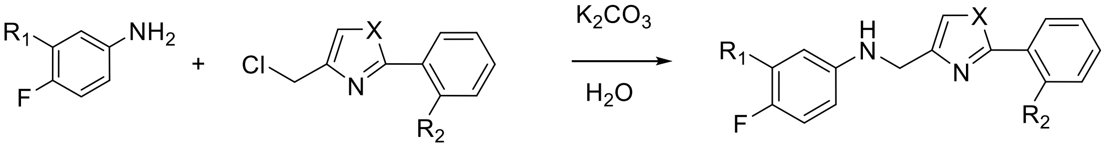

ORM-11863: R_1 = F; R_2 = Cl; X = S
ORM-11298: R_1 = Cl; R_2 = H; X = O

Methods

All solvents and chemicals were used as purchased without further purification. Column chromatography was carried out on Silica gel 60 (0.040 - 0.063 mm) or using CombiFlash Companion equipment with RediSep® Rf normal phase columns (Teledyne Isco). ^{1}H NMR spectra were recorded on a Bruker Avance 400 spectrometer, with DMSO-d_6 as solvent and tetramethylsilane as an internal reference. MS analysis was performed using Polaris Q Ion Trap mass spectrometer GC/MS system. The purity was determined by HPLC with Agilent 1 100 Series HLPC equipped with UV detector. Purities of the final compounds were all over 96%.

General procedure A (Scheme 1):
In a round bottom flask, the aniline derivative (1 equivalent), the furan carbaldehyde derivative (1 equivalent), p-toluenesulfonic acid (5 mol-%) and toluene were added. The reaction mixture was refluxed using a Dean-Stark apparatus until the reaction was complete. Solvents were evaporated, and dry ethanol and $NaBH_4$ (2 equivalent) were subsequently added to the flask. The reaction mixture was stirred at room temperature until completion of the reaction. The pH of the reaction mixture was made acidic using 1 M hydrochloric acid solution. The acidic reaction mixture was extracted with ethyl acetate, and the combined organic phases were dried with Na_2S0_4 and solvents were evaporated.

General procedure B (Scheme 2):
To a round bottom flask, the aniline derivative (1 - 1.5 eq), the phenyl oxazole derivative (1 equivalent), the potassium carbonate (0.5 - 1 equivalent) and distilled water were added. The reaction mixture was refluxed until completion of the reaction. The reaction mixture was then allowed to cool to room temperature and extracted with ethyl acetate. The combined organic phase was washed with brine and dried over Na_2S0_4. Solvents were evaporated.

Starting materials:
All starting materials except 5-(pyridin-2-yl)furan-2-carbaldehyde (Enamine Building Blocks) are available from Sigma-Aldrich.

Figure S3. ORM-120407

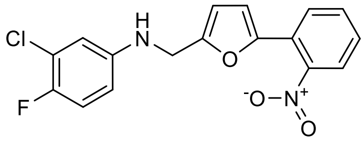

3-chloro-4-fluoro-N-((5-(2-nitrophenyl)furan-2-yl)methyl)aniline

The title compound was prepared according to the general procedure A using 3-chloro-4-fluoroaniline (1.00 g, 6.87 mmol), 5-(2-nitrophenyl)furan-2-carbaldehyde (1.492 g, 6.87 mmol), *p*-toluenesulfonic acid (36.4 mg, 0.34 mmol) and toluene (100 ml). The amine reduction was performed using dry ethanol (80 ml) and $NaBH_4$ (0.520 g, 13.74 mmol). The product was purified by filtrating the reaction mixture through a silica bed using ethyl acetate – heptane 1:1 mixture as a solvent. The product was converted to HCl salt. Isolated yield 507 mg. MS (EI, 70 eV): m/z (%) 202 (100), 128 (24), 346 (16), 203 (13), 348 (5).

^{1}H NMR (DMSO-d_6) δ: 7.79-7.87 (m, 2H), 7.68-7.74 (m, 1H), 7.54-7.57 (m, 1H), 7.08-7.14 (m, 1H), 6.76-6.79 (m, 2H), 6.59-6.63 (m, 1H), 6.46 (d, 1H), 4.26 (s, 2H).

Figure S4. ORM-11023

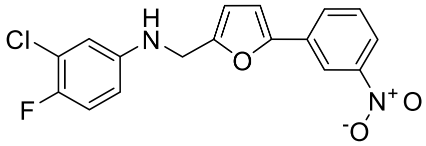

3-chloro-4-fluoro-N-((5-(3-nitrophenyl)furan-2-yl)methyl)aniline

The title compound was prepared according to the general procedure A using 3-chloro-4-fluoroaniline (0.20 g, 1.37 mmol), 5-(3-nitrophenyl)furan-2-carbaldehyde (0.298 g, 1.37 mmol), *p*-toluenesulfonic acid (7.3 mg, 0.069 mmol) and toluene (40 ml). The amine reduction was performed using dry ethanol (30 ml) and $NaBH_4$ (0.104 g, 2.75 mmol). The product was converted to HCl salt and triturated with methanol. Isolated yield 150 mg. MS (EI, 70 eV): m/z (%) 202 (100), 156 (38), 203 (13), 128 (11) 346 (10), 348 (3).

^{1}H NMR (DMSO-d_6) δ: 8.39-8.41 (m, 1H), 8.08-8.11 (m, 2H), 7.67-7.73 (m, 1H), 7.08-7.17 (m, 2H), 6.81-6.84 (m, 1H), 6.63-6.69 (m, 1H), 6.49 (d, 1H), 4.35 (s, 2H).

Figure S5. ORM-11024

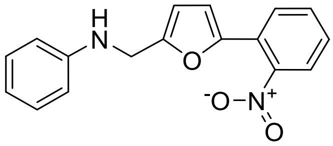

N-((5-(2-nitrophenyl)furan-2-yl)methyl)aniline

The title compound was prepared according to the general procedure A using aniline (0.20 g, 2.15 mmol), 5-(2-nitrophenyl)furan-2-carbaldehyde (0.467 g, 2.15 mmol), *p*-toluenesulfonic acid (11.4 mg, 0.11 mmol) and toluene (40 ml). The amine reduction was performed using dry ethanol (30 ml) and $NaBH_4$ (0.163 mg, 4.30 mmol). The product was purified by preparative TLC chromatography (Merck, PLC Silica gel 60 F_{254}, 2 mm)) using ethyl acetate and heptane as eluent.The product was converted to HCl salt. Isolated yield 70 mg. MS (EI, 70 eV): m/z (%) 202 (100), 294 (37), 128 (29), 203 (13), 156 (11), 295 (8).

^{1}H NMR (DMSO-d_6) δ: 7.79-7.87 (m, 2H), 7.68-7.74 (m, 1H), 7.52-7.57 (m, 1H), 7.09-7.14 (m, 2H), 6.71-6.78 (m, 3H), 6.62-6.67 (m, 1H), 6.45 (d, 1H), 4.29 (s, 2H).

Figure S6. ORM-11165

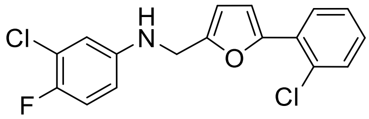

3-chloro-N-((5-(2-chlorophenyl)furan-2-yl)methyl)-4-fluoroaniline

The title compound was prepared according to the general procedure A using 3-chloro-4-fluoroaniline (0.11 g, 0.73 mmol), 5-(2-chlorophenyl)furan-2-carbaldehyde (0.15 g, 0.73 mmol), p-toluenesulfonic acid (3.8 mg, 0.037 mmol) and toluene (40 ml). The amine reduction was performed using dry ethanol (30 ml) and NaBH4 (55.2 mg, 1.46 mmol). The product was converted to HCl salt. Isolated yield 210 mg. MS (EI, 70 eV): m/z (%) 191 (100), 193 (32), 128 (29), 127 (15), 192 (13), 129 (4), 194 (4), 335 (5), 337 (3).

^{1}H NMR (DMSO-d_6) δ: 7.79-7.81 (m, 1H), 7.52-7.55 (m, 1H), 7.40-7.44 (m, 1H), 7.30-7.34 (m, 1H), 7.10-7-15 (m, 1H), 7.06 (d, 1H), 6.81-6.84 (m, 1H), 6.64-6.67 (m, 1H), 6.48 (d, 1H), 4.34 (s, 2H).

Figure S7. ORM-11190

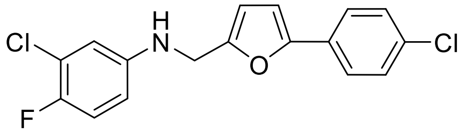

3-chloro-N-((5-(4-chlorophenyl)furan-2-yl)methyl)-4-fluoroaniline

The title compound was prepared according to the general procedure A using 3-chloro-4-fluoroaniline (0.11 g, 0.73 mmol), 5-(4-chlorophenyl)furan-2-carbaldehyde (0.15 g, 0.73 mmol), *p*-toluenesulfonic acid (3.8 mg, 0.037 mmol) and toluene (40 ml). The amine reduction was performed using dry ethanol (30 ml) and $NaBH_4$ (55.2 mg, 1.46 mmol). The product was converted to HCl salt. The product was purified by filtrating the reaction mixture through a silica bed using ethyl acetate and heptane as eluent. Isolated yield 160 mg. MS (EI, 70 eV): m/z (%) 191 (100), 193 (33), 128 (24), 127 (15), 192 (13), 335 (6), 194 (4), 337 (3).

^{1}H NMR (DMSO-d_6) δ: 7.65-7.68 (m, 2H), 7.45-7.48 (m, 2H), 7.09-7.14 (m, 1H), 6.92 (d, 1H), 6.79-6.82 (m, 1H), 6.62-6.66 (m, 1H), 6.43 (d, 1H), 4.30 (s, 2H).

Figure S8. ORM-11217

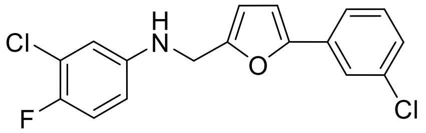

3-chloro-N-((5-(3-chlorophenyl)furan-2-yl)methyl)-4-fluoroaniline

The title compound was prepared according to the general procedure A using 3-chloro-4-fluoroaniline (0.11 g, 0.73 mmol), 5-(3-chlorophenyl)furan-2-carbaldehyde (0.15 g, 0.73 mmol), *p*-toluenesulfonic acid (3.8 mg, 0.037 mmol) and toluene (40 ml). The amine reduction was performed using dry ethanol (30 ml) and $NaBH_4$ (55.0 mg, 1.46 mmol). The product was purified by column chromatography using ethyl acetate and heptane as eluent.The product was converted to HCl salt. Isolated yield 38 mg. MS (EI, 70 eV): m/z (%) 191 (100), 193 (32), 128 (31), 127 (19), 192 (12), 335 (6), 129 (6), 194 (5), 337 (4).

^{1}H NMR (DMSO-d_6)δ: 7.69-7.70 (m, 1H), 7.60-7.63 (m, 1H), 7.41-7.45 (m, 1H), 7.30-7.33 (m, 1H), 7.10-7.15 (m, 1H), 7.00 (d, 1H), 6.82-6.85 (m, 1H), 6.65-6.68 (m, 1H), 6.44 (d, 1H), 4.32 (s, 2H).

Figure S9. intermediate of ORM-11372

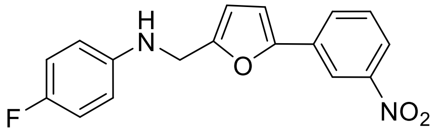

4-Fluoro-N-((5-(3-nitrophenyl)furan-2-yl)methyl)aniline

The title compound was prepared according to the general procedure A using 4-fluoroaniline (2.56 g, 23 mmol), 5-(3-nitrophenyl)furan-2-carbaldehyde (5.00 g, 23 mmol), p-toluenesulfonic acid (0.219 g, 1.1 mmol) and toluene (150 ml). The amine reduction was performed using dry ethanol (100 ml) and NaBH4 (1.74 g, 46 mmol). The product was purified by triturating with heptane. Isolated yield 7.18 g. MS (EI, 70 eV): m/z (%) 202 (100), 156 (51), 128 (20), 312 (18), 203 (13), 157 (6), 127 (6), 313 (3).

^{1}H NMR (DMSO-d_6) δ: 8.40-8.41 (m, 1H), 8.08-8.10 (m, 2H), 7.68-7.72 (m, 1H), 7.16 (d, 1H), 6.91-6.95 (m, 2H), 6.67-6.70 (m, 2H), 6.47 (d, 1H), 6.11-6.13 (m, 1H), 4.32 (d, 2H).

Figure S10 ORM-11372

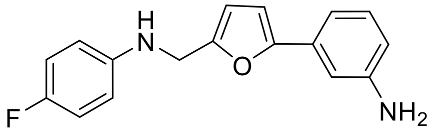

N-((5-(3-aminophenyl)furan-2-yl)methyl)-4-fluoroaniline

Example 23 in the patent application (Otsomaa L., 2018)
4-Fluoro-N-((5-(3-nitrophenyl)furan-2-yl)methyl)aniline (0.66 g, 2.1 mmol) was dissolved in acetic acid (150 ml). Zinc (2.75 g, 42 mmol) was added in small portions to the solution and the reaction mixture was stirred at room temperature until completion of the reaction. The reaction mixture was filtered through a pad of Celite®, which was washed with acetic acid and methanol. The filtrate was evaporated. The evaporation residue was dissolved in ethyl acetate and washed with 1 M sodium hydroxide. The product was purified by flash column chromatography using ethyl acetate and toluene as eluent. Isolated yield 466 mg. MS (EI, 70 eV): m/z (%) 172 (100), 173 (13), 282 (8).

^{1}H NMR (DMSO-d_6) δ: 7.65-7.71 (m, 1H), 7.60 (s, 1H), 7.47-7.55 (m, 1H), 7.25-7.30 (m, 1H), 7.01-7.08 (m, 2H), 6.87-6.97 (m, 3H), 6.50 (d, 1H), 4.40 (s, 2H).

Figure S11. ORM-11817

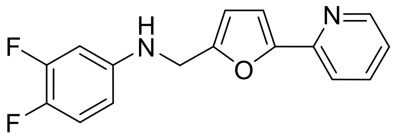

3,4-difluoro-N-((5-(pyridin-2-yl)furan-2-yl)methyl)aniline

Example 30 in the patent application [15]
The title compound was prepared according to the general procedure A using 3,4-difluoroaniline (0.924 g, 7.16 mmol), 5-(pyridin-2-yl)furan-2-carbaldehyde (1.24 g, 7.16 mmol), p-toluenesulfonic acid (68 mg, 0.36 mmol) and toluene (100 ml). The amine reduction was performed using dry ethanol (80 ml) and NaBH4 (0.542 g, 14.32 mmol). The product was purified by flash column chromatography using ethyl acetate and heptane as eluent. Isolated yield 1.365 g. MS (EI, 70 eV): m/z (%) 158 (100), 78 (15), 286 (14), 159 (11), 106 (11), 287 (3).

^{1}H NMR (DMSO-d_6) δ: 8.52-8.57 (m, 1H), 7.80-7.87 (m, 1H), 7.64-7.69 (m, 1H), 7.23-7.29 (m, 1H), 7.07-7.16 (m, 1H), 7.03 (d, 1H), 6.62-6.70 (m, 1H), 6.43-6.51 (m, 2H), 6.37-6.43 (m, 1H), 4.33 (d, 2H).

Figure S12. ORM-11875

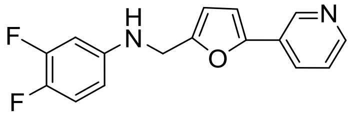

3,4-difluoro-N-((5-(pyridin-3-yl)furan-2-yl)methyl)aniline

Example 29 in the patent application [15]
The title compound was prepared according to the general procedure A using 3,4-difluoroaniline (0.381 g, 2.95 mmol), 5-(pyridin-3-yl)furan-2-carbaldehyde (0.511 g, 2.95 mmol), p-toluenesulfonic acid (28 mg, 0.15 mmol) and toluene (60 ml). The amine reduction was performed using dry ethanol (40 ml) and NaBH4 (0.223 g, 5.90 mmol). The product was purified by flash column chromatography using ethyl acetate and heptane as eluent. Isolated yield 324 mg.

^{1}H NMR (DMSO-d_6) δ: 8.89- 8.92 (m, 1H), 8.44-8.48 (m, 1H), 7.98-8.03 (m, 1H), 7.41-7.46 (m, 1H), 7.08-7.16 (m, 1H), 7.03 (d, 1H), 6.63-6.71 (m, 1H), 6.45-6.50 (m, 2H), 6.32-6.42 (m, 1H), 4.32 (d, 2H).

Figure S13. ORM-11863

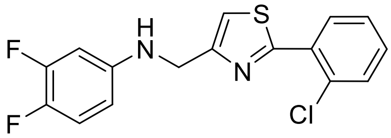

N-((2-(2-chlorophenyl)thiazol-4-yl)methyl)-3,4-difluoroaniline

The title compound was prepared according to the general procedure B using 3,4-difluoroaniline (0.531 g, 4.11 mmol), 4-(chloromethyl)-2-(2-chlorophenyl)-1,3-thiazole (0.67 g, 2.74 mmol), potassium carbonate (0.19 g, 1.37 mmol) and water (10 ml). The product was purified by preparative TLC chromatography (PLC Silica gel 60 F_{254}, 2 mm) using ethyl acetate and heptane as eluent.The product was converted to HCl salt. Isolated yield 50 mg.

^{1}H NMR (DMSO-d_6) δ: 8.18-8.22 (m, 1H), 7.62-7.66 (m, 2H), 7.48-7.53 (m, 2H), 7.07-7.15 (m, 1H), 6.62-6.68 (m, 1H), 6.43-6.46 (m, 1H), 4.43 (s, 2H).

Figure S14. ORM-11298

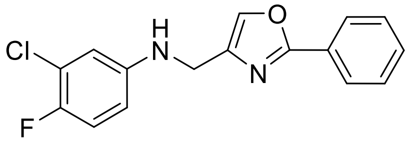

3-chloro-4-fluoro-N-((2-phenyloxazol-4-yl)methyl)aniline

Example 2 in patent application [15]
The title compound was prepared according to the general procedure B using 3-chloro-4-fluoroaniline (16.5 g, 114 mmol), 4-(chloromethyl)-2-phenyloxazole (22 g, 114 mmol), K_2CO_3 (7.85 g, 57 mmol) and water (500 ml). The product was purified by flash column chromatography using ethyl acetate and heptane as eluent. The product was further purified by recrystallization from ether/heptane and converted to HCl salt. Isolated yield 8.06 g. MS (EI, 70 eV): m/z (%) 302 (100), 103 (64), 158 (56), 303 (38), 304 (37), 131 (36), 104 (34), 301 (29), 159 (24), 105 (15), 305 (11).

^{1}H NMR (DMSO-d_6) δ: 8.07 (s, 1H), 7.94-7.99 (m, 2H), 7.51-7.56 (m, 3H), 7.08-7.15 (m, 1H), 6.78-6.82 (m, 1H), 6.61-6.66 (m, 1H), 4.20 (s, 2H).

Figure S15

The effect of ORM-11372 on K_V11.1 current. A, ORM-11372 concentration dependently inhibited I_{hERG} with IC_{50} values of 19.2 and 10.0 µM when determined respectively with automated (0.3 µM, n = 4 cells; 1 µM, n = 5; 3 µM, n = 8; 10, µM, n = 8; 30 µM, n = 8) and manual (n = 4 cells at each concentration) patch-clamp. B-D, Voltage dependency of ORM-11372 block of I_{hERG}. On the automated patch-clamp, single whole-cell currents (A) were evoked by the voltage protocol shown inset in control conditions and then in the presence of 20 µM ORM-11372 (close to the IC_{50}). For clarity sweeps are shown for every 20 mV change. C, Current-voltage relationship of the peak tail currents in control conditions and in the presence of ORM-11372 (n=7 cells). The peak tail current amplitudes were normalized to the control value at +60 mV for that cell. The data was fitted with a Boltzmann equation (control: $V_{½}$ = -7.1 ± 1.0 mV, k = 6.9 ± 0.3 mV; ORM-11372 $V_{½}$ = -18.3 ± 0.7 mV, k = 5.5 ± 0.1 mV; n = 7; for both $V_{½}$ and k p<0.05 Student's *t*-test). D, Voltage-dependence of tail current inhibition (n=7 cells). * Significant difference in relative current ($I_{ORM}/I_{Control}$) from that obtained at +60 mV (p<0.05 One way ANOVA followed by Dunnett test).

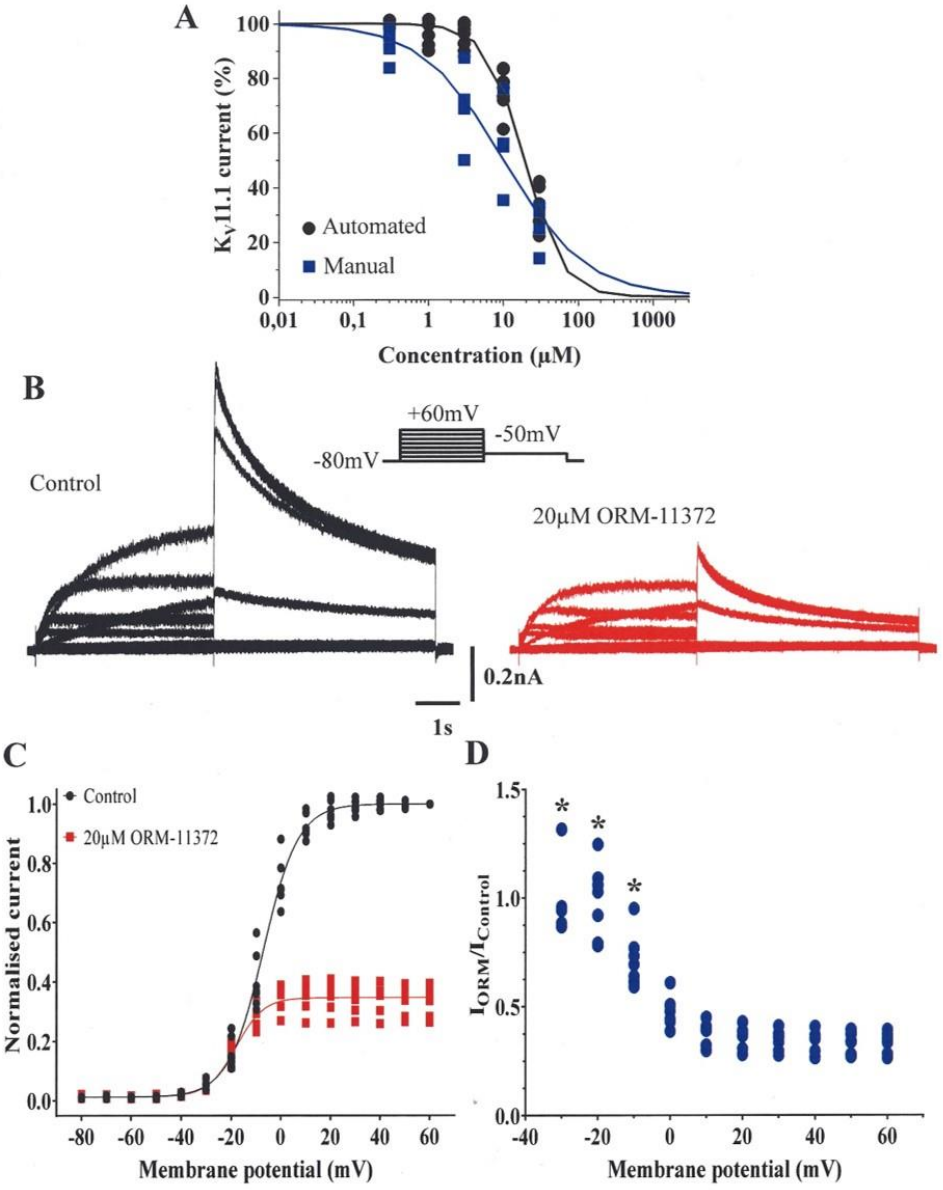

Figure S16

The effect of ORM-11372 on the inward calcium current (I_{CaL}) in isolated rat cardiomyocytes. I_{CaL} current was evoked by 400 ms-long depolarizing voltage pulses to various test potentials, ranging from -35 mV to 55 mV. The holding potential was -80 mV. A short prepulse to -40 mV served to inactivate the Na^{+} current (the top part of the panel A). Examples of originals currents are shown in the lower part of panel A, before and after 1 µM ORM-11372 exposure in an isolated rat myocyte. Panels B and C illustrate the current-voltage relation (I-V curves) of I_{CaL} in the absence and presence of 1 µM and 10 µM ORM-11372 (panels B and C, respectively). 1 µM ORM-11372 (n=3, 5 cells originated from 3 rats) did not change, while 10 µM ORM-11372 (n=4; 6 cells originated from 4 rats) moderately decreased calcium current at various test potentials. Values are means ± standard errors of the means.

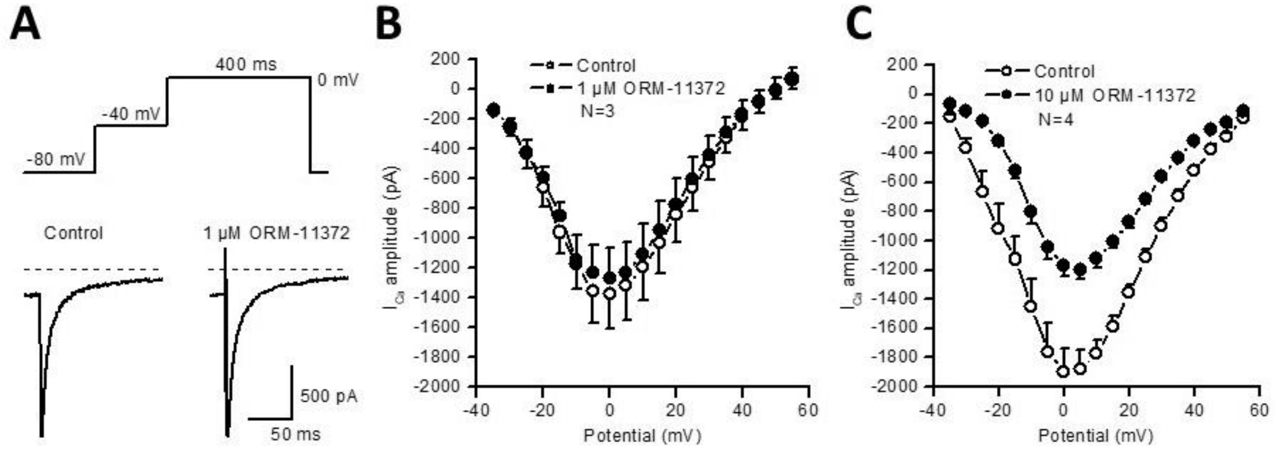

Figure S17

Mean % changes in APD_{90} (1 Hz, the panel A; 2 Hz, the panel D), APD_{50} (1 Hz, B; 2 Hz, E) & APD_{30} (2 Hz, C; 2 Hz, F) induced by addition of ORM-11372 and after exposure to dofetilide (n=2; 2 trabeculae from each heart). Dof, Dofetilide; H: Heart; T: Trabecula. The abbreviation n refers to number of human hearts.

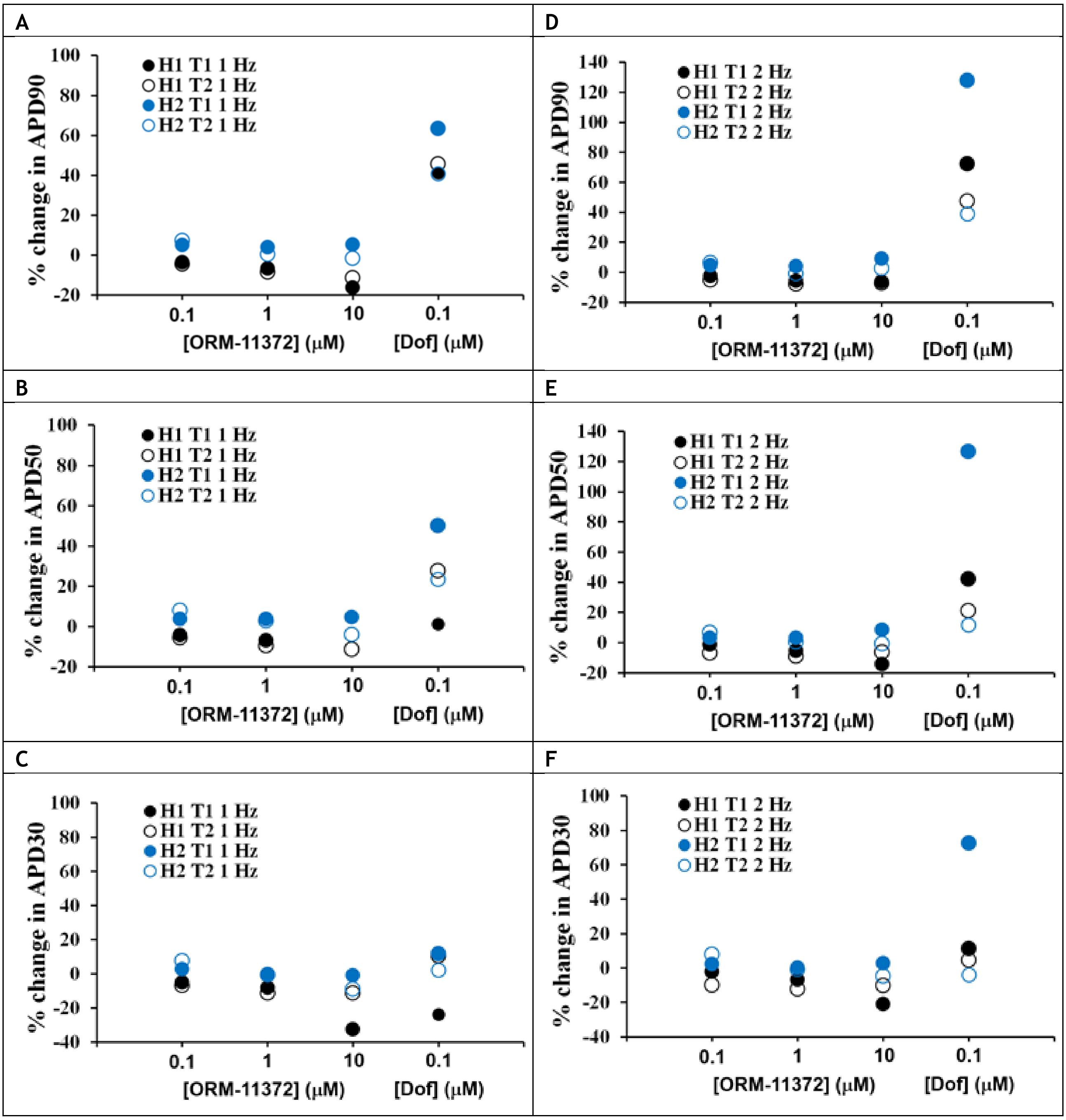

Figure S18

Effect of ORM-11372 (A: 0.1 µM; B: 0.3 µM) on human iPS cardiomyocyte action potentials. A left, Action potentials recorded in current-clamp at baseline (black trace), during exposure to ORM-11372 (red trace) and during wash off (grey trace). The dashed line denotes 0 mV. On the right is plotted, for the individual cardiomyocytes (n = 4), the action potential duration (APD) at 30, 50 and 90 % repolarisation in 0.1 µM ORM-11372 as the percentage of their baseline values. B, Action potentials and APDs recorded with voltage sensitive dye at baseline (black trace) and after 30 min treatment with either vehicle (0.1 % DMSO; blue trace and circles) or 0.3 µM ORM-11372 (red trace and squares). The APDs at 30, 50 and 90 % repolarisation are plotted on the right for 6 wells in each condition, the means are significantly different between the vehicle and ORM-11372 (*p<0.05 unpaired Student's *t*-test).

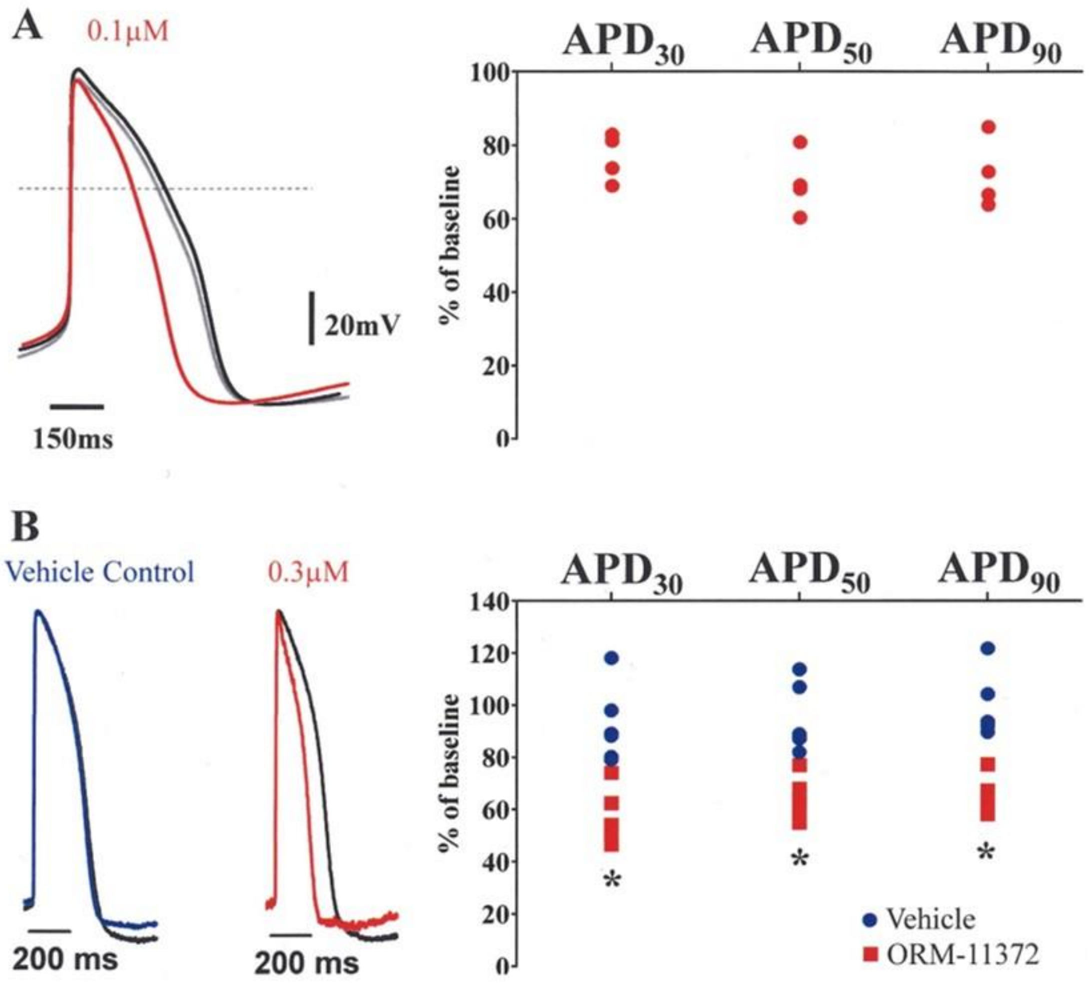

Figure S19

Mean % changes in triangulation (A, B) and changes in STV (C, D) induced by addition of ORM-11372 and after exposure to dofetilide at 1 and 2 Hz (n=2; 2 trabeculae from each heart). V: Vehicle; Dof, Dofetilide; H: Heart; T: Trabecula. The abbreviation n refers to number of human hearts.

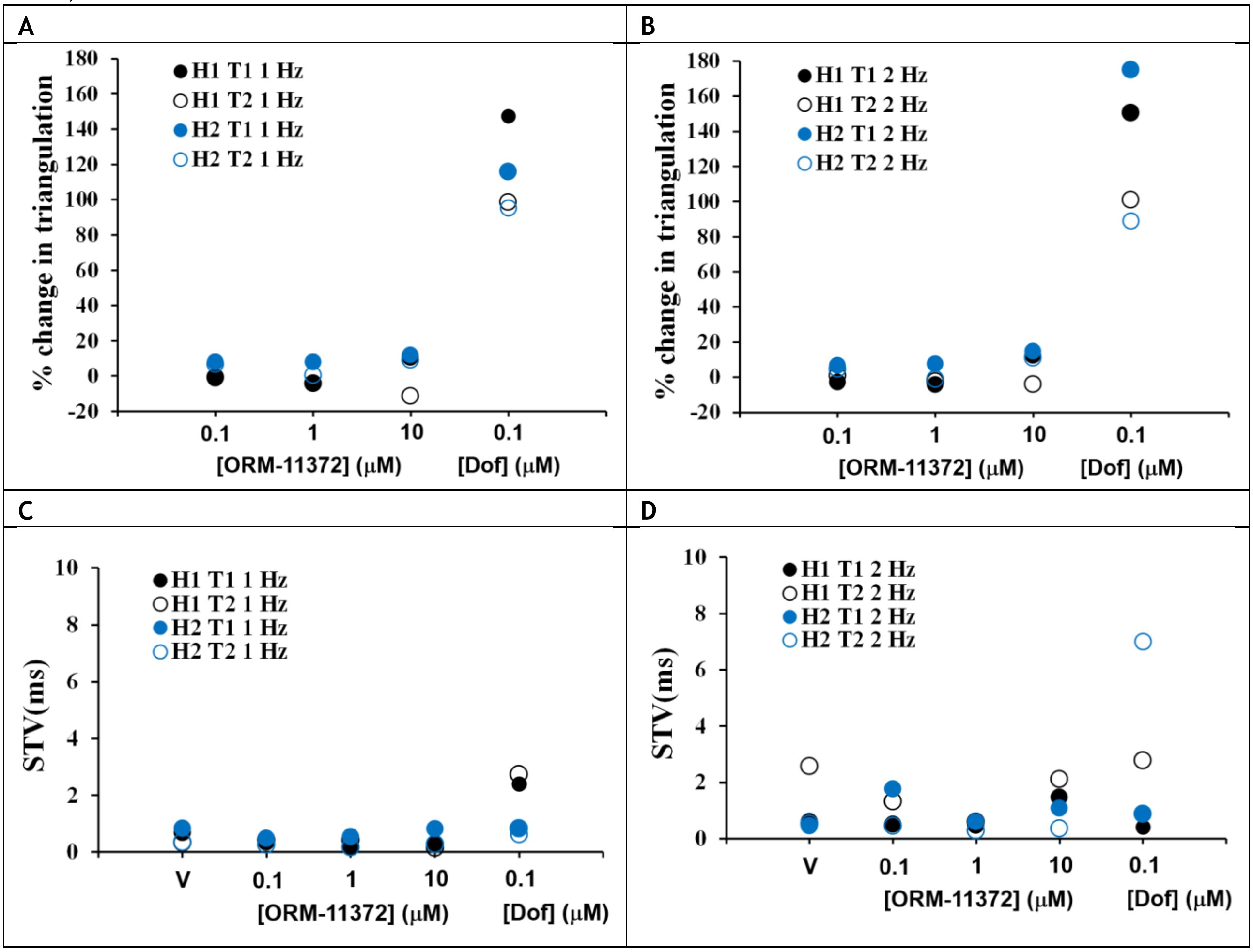

In vivo haemodynamics in healthy rats

Materials and Methods

Experimental Animals and General Study Protocol

Rat was selected to the study as it is the most widely used rodent species in the toxicology studies performed for the small molecules during the drug development. However, functional similarity of Ca^{2+} handling proteins (including NCX) of rat and human hearts is low. The balance of Ca^{2+} circulation in the rabbit heart is more similar like human, hence rabbit was selected as a nonrodent species. All animal experiments were made according to European Community Guidelines for the use of experimental animals and approved by Finnish National Animal Experiment Board. All studies complied with the ARRIVE guidelines, (NC3Rs Reporting Guidelines Working Group, 2010).

Specific pathogen free animals were housed in half-barrier rooms where special protecting clothing for personnel was required. Animal rooms were cleaned regularly 3 times per week and cages and bottles were

changed regular intervals once a week. Temperature was maintained at 22 ± 2°C and humidity at 55 ± 15%. Light-dark cycle was lights on from 06.00 to 20.00h.

Male Sprague-Dawley rats (Harlan, Netherlands B.V.) were housed in polycarbonate cages (Makrolon® IV with stainless steel wire mesh lids). Maximally 5 rats were housed per cage with aspen chip bedding (Tapvei Ky, Kaavi, Finland). Rats had free access to water (twice filtered, Espoon Vesi) and rodent diet (SDS RM1 (P) SQC pelleted diet, Special Diet Services Ltd, Witham, England). Acclimation period before experiments was at least 5 days. Healthy 10-12 weeks aged weighing 300-400 g rats were randomized to ORM-11372, dobutamine or vehicle group.

At the end of the haemodynamic experiments, rats were terminated with an overdose of pentobarbital.

Anesthesia and ventilation

Rats were anaesthetised with isoflurane (2.25-2.5 %, Baxter) in carbogen (95 % O_2 and 5 % CO_2) and nitrous oxide (1:1) using a small rodent ventilator (Ugo Basile 7025, ~10 ml/kg, 60 strokes/min). The rats were infused with 0.9 % NaCl in carotid vein at the stabilization and baseline level. Animals were placed on a heating table (+38°C). Tracheas were cannulated.

Hemodynamic measurements

Systemic blood (SP) and left ventricular pressures (LVP) were measured with pressure transducers (BP: Isotec, Hugo Sachs Elektronic, Germany; LVP: Mikro-Tip transducer SPR-249, Millar Instruments, USA). The signal was amplified (DC-bridge amplifier type 660, Hugo Sachs Elektronik, Germany), digitised (I/O connector block type SCB-68, National Instruments, USA), recorded and analysed (IHME 1.0.9, Fision Ltd, Finland). Left ventricular inotropic effect (LV +dP/dt_{max}) and relaxation (LV -dP/dt_{min}) and heart rate (HR) were analysed from LVP signal.

Blood sampling for the evaluation of plasma levels of the drug

At the end of each infusion, the blood samples (300-500 µl) were taken into chilled EDTA polypropylene tube (CapiJect®, Terumo) and centrifuged (4000 rpm, 10 min, at 4°C). Plasma was immediately frozen in polypropylene tubes and stored at –20°C. ORM-11372 and dobutamine were extracted from plasma samples using a liquid–liquid extraction and analysed using liquid chromatography–tandem mass spectrometry (Agilent Technologies series 1100 liquid chromatographic system and a Sciex LC-MS/MS API 4000 mass spectrometry). The lower limit of quantification for total plasma concentrations were 32.0 and 5.00 ng/ml from total for ORM-11372 and dobutamine, respectively. Plasma protein binding for ORM-11372 was assessed using TRANSIL High Sensitivity Binding Assay was used according to the manufacturer's instructions (Sovicell, GmbH, Germany).

Results

ORM-11372 was also shown to have unique pharmacological profile compared to dobutamine. Dobutamine increases contractility and heart rate, both effects can lead to increased oxygen and energy consumption. The effects of dobutamine on blood pressure varies partially due to the use of racemate as active ingredient in the pharmaceutical formulation. (-)-dobutamine is α_1-agonist and therefore causes increases in cardiac output and peripheral resistance. (+)-dobutamine acts via β_1- and β_2-adrenoceptors which leads to increase in heart rate and reduction in blood pressure [16].

ORM-11372 and dobutamine induced 10±2 and 16±3% positive inotropic effect at the free plasma concentration of 4±0.3 and 16±0.8 nM, respectively (Figure S19A). 25% positive inotropic was achieved by ORM-11372 and dobutamine at the free plasma concentration of 36±1.4 and 25±1.6 nM, respectively. The maximum inotropic effect without any other effects for ORM-11372 was 34±5 % at 92±8 nM. ORM-11372 and dobutamine induced the absolute maximum positive inotropic effects were 44 and 59 % at 351±92 and 199±17 nM, respectively.

ORM-11372 tended to improve relaxation by 7.5±4.5% at 36 nM. However, at high concentration (≥240 nM) ORM-11372 impaired relaxation (Figure S19B). Dobutamine had no effects on relaxation at the free plasma concentrations between 16 to 199 nM.

ORM-11372 had no effect on heart rate up to 240 nM free plasma concentration. At higher concentrations there was -7 to -12% decrease in heart rate (Figure S19C). Dobutamine increased heart rate from 15±5% up to 25±7% at the free plasma concentration from 96 to 848 nM.

In vitro 36 nM concentration of ORM-11372 inhibits 67% of NCX 1.1 current. The absolute maximum positive inotropic effects were 44% (at 351 nM, 90% NCX inhibition) and 59% (at 199 nM) for ORM-11372 and dobutamine, respectively. ORM-11372 did not affect left ventricular relaxation or systolic blood pressure from 4 nM up to 92 nM of free plasma concentration corresponding in vitro NCX 1.1 current inhibitions from 34% to 79%. ORM-11372 had no effects on heart rate up to 240 nM. Dobutamine increased dose dependently heart rate starting at free plasma concentration 96 nM and higher concentrations.

Figure S20 Haemodynamics in healthy rats

Effects of ORM-11372 and dobutamine on haemodynamics in isoflurane anesthetized 10-12 weeks aged male Sprague-Dawley rats (Harlan, Netherlands B.V.) weighing 300-400 g. Shown are mean ± SEM and individual effects on the left ventricular contractility (LV+dP/dt$_{max}$, panel A), relaxation (LV-dP/dt$_{max}$, panel B), heart rate (panel C), and systolic arterial blood pressure (SAP, paned D). ORM-11372 (n=4) and dobutamine (n=4) ascending infusion doses were 17, 83, 167, 333, 500, 667, and 833 µg/kg/min (20 min/dose) and 0.05, 0.1, 0.5, 1, 2, 3, and 4 µg/kg/min (15 min/dose), respectively. Statistics is not calculated due to the number of experiments is 4.

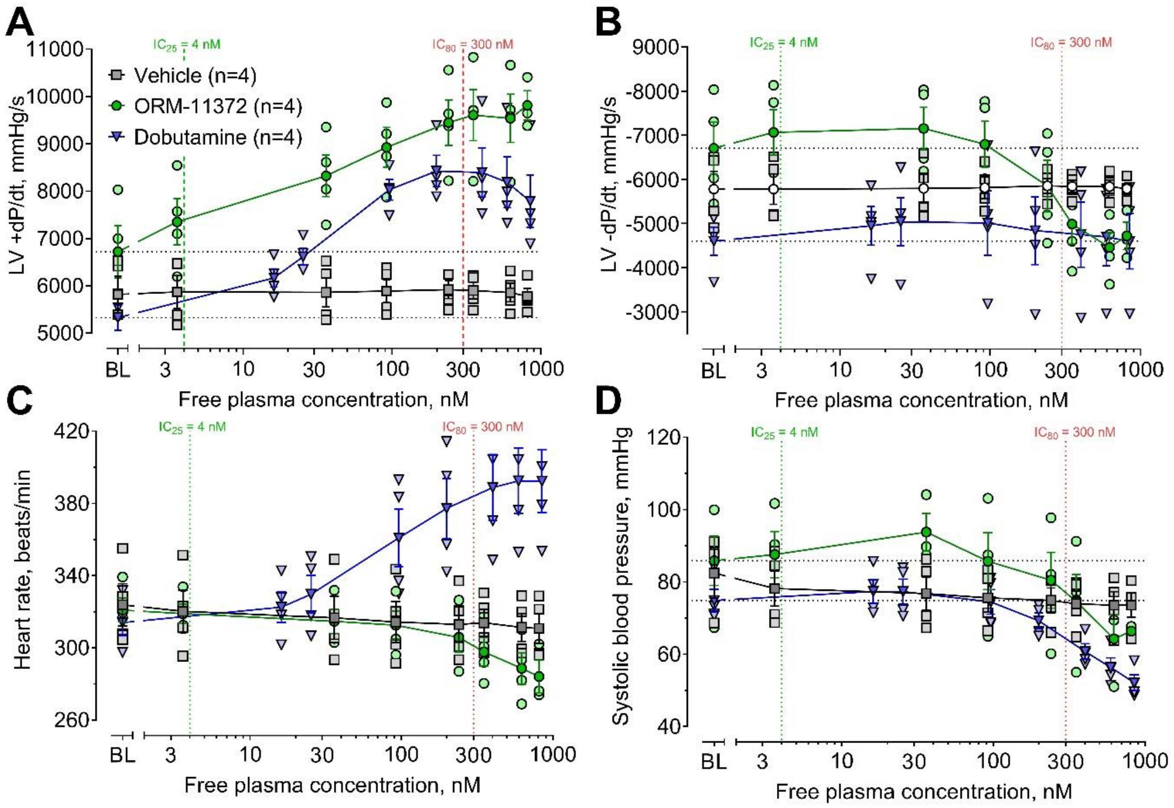

Figure S20. Myocardial infarction model in rats.

In panel A, examples of histopathological slices at apex, mid part, and base level of hearts of sham and myocardial infarction (MI) male rats (Sprague-Dawley, panel A). In the panel B, are shown mean (± SEM) and individual infarct sizes (n=6). Unpaired t-test. *p<0.05.

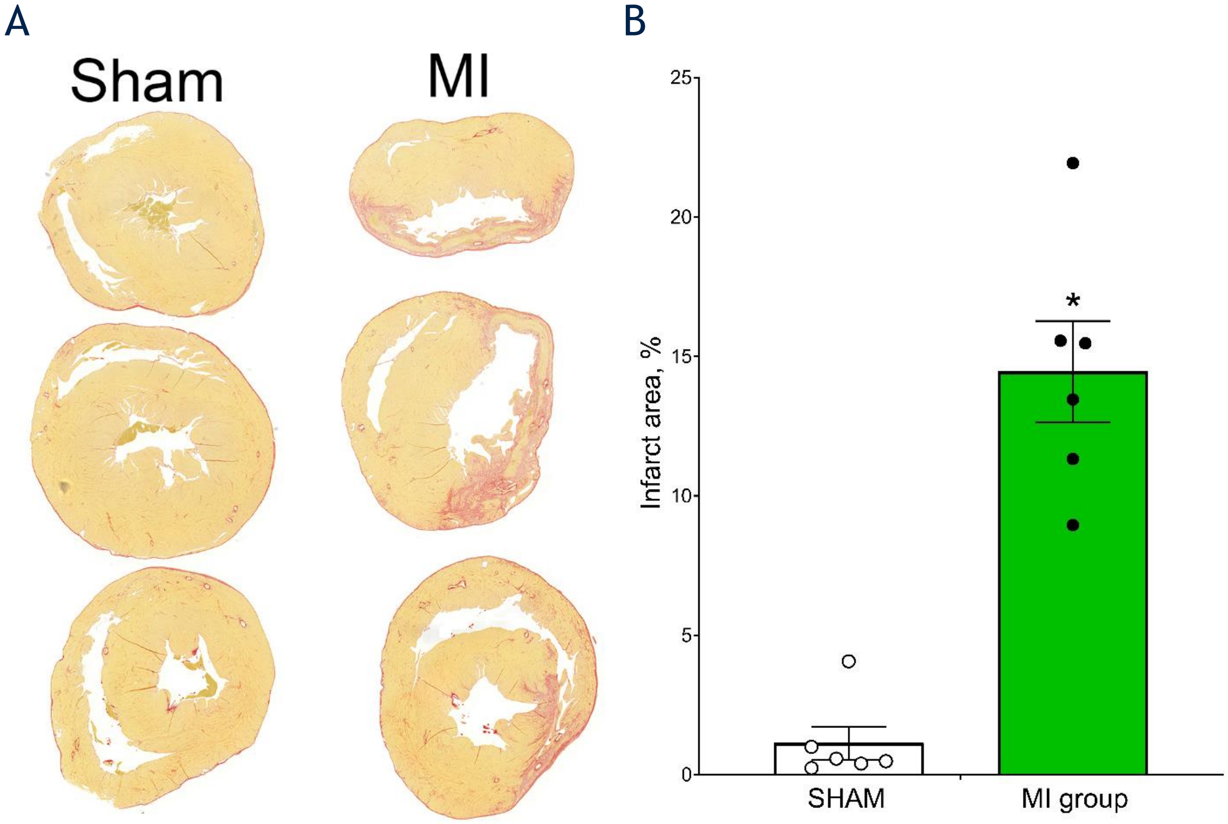

Reference

1. Farkas AS, Acsai K, Nagy N, Toth A, Fulop F, Seprenyi G, Birinyi P, Nanasi PP, Forster T, Csanady M, Papp JG, Varro A and Farkas A. Na(+)/Ca(2+) exchanger inhibition exerts a positive inotropic effect in the rat heart, but fails to influence the contractility of the rabbit heart. *Br J Pharmacol*. 2008;154:93-104.
2. Cheng H, Zhang Y, Du C, Dempsey CE and Hancox JC. High potency inhibition of hERG potassium channels by the sodium-calcium exchange inhibitor KB-R7943. *Br J Pharmacol*. 2012;165:2260-73.
3. Birinyi P, Acsai K, Banyasz T, Toth A, Horvath B, Virag L, Szentandrassy N, Magyar J, Varro A, Fulop F and Nanasi PP. Effects of SEA0400 and KB-R7943 on Na+/Ca2+ exchange current and L-type Ca2+ current in canine ventricular cardiomyocytes. *Naunyn Schmiedebergs Arch Pharmacol*. 2005;372:63-70.
4. Iwamoto T, Inoue Y, Ito K, Sakaue T, Kita S and Katsuragi T. The exchanger inhibitory peptide region-dependent inhibition of Na+/Ca2+ exchange by SN-6 [2-[4-(4-nitrobenzyloxy)benzyl]thiazolidine-4-carboxylic acid ethyl ester], a novel benzyloxyphenyl derivative. *Mol Pharmacol*. 2004;66:45-55.
5. Iwamoto T and Shigekawa M. Differential inhibition of Na+/Ca2+ exchanger isoforms by divalent cations and isothiourea derivative. *Am J Physiol*. 1998;275:C423-30.
6. Tanaka H, Nishimaru K, Aikawa T, Hirayama W, Tanaka Y and Shigenobu K. Effect of SEA0400, a novel inhibitor of sodium-calcium exchanger, on myocardial ionic currents. *Br J Pharmacol*. 2002;135:1096-100.
7. Jost N, Nagy N, Corici C, Kohajda Z, Horvath A, Acsai K, Biliczki P, Levijoki J, Pollesello P, Koskelainen T, Otsomaa L, Toth A, Papp JG, Varro A and Virag L. ORM-10103, a novel specific inhibitor of the Na+/Ca2+ exchanger, decreases early and delayed afterdepolarizations in the canine heart. *Br J Pharmacol*. 2013;170:768-78.
8. Kohajda Z, Farkas-Morvay N, Jost N, Nagy N, Geramipour A, Horvath A, Varga RS, Hornyik T, Corici C, Acsai K, Horvath B, Prorok J, Ordog B, Deri S, Toth D, Levijoki J, Pollesello P, Koskelainen T,

Otsomaa L, Toth A, Baczko I, Lepran I, Nanasi PP, Papp JG, Varro A and Virag L. The Effect of a Novel Highly Selective Inhibitor of the Sodium/Calcium Exchanger (NCX) on Cardiac Arrhythmias in In Vitro and In Vivo Experiments. *PLoS One*. 2016;11:e0166041.
9. Iwamoto T and Kita S. YM-244769, a novel Na+/Ca2+ exchange inhibitor that preferentially inhibits NCX3, efficiently protects against hypoxia/reoxygenation-induced SH-SY5Y neuronal cell damage. *Mol Pharmacol*. 2006;70:2075-83.
10. Satoh H, Ginsburg KS, Qing K, Terada H, Hayashi H and Bers DM. KB-R7943 block of Ca(2+) influx via Na(+)/Ca(2+) exchange does not alter twitches or glycoside inotropy but prevents Ca(2+) overload in rat ventricular myocytes. *Circulation*. 2000;101:1441-6.
11. Armoundas AA, Hobai IA, Tomaselli GF, Winslow RL and O'Rourke B. Role of sodium-calcium exchanger in modulating the action potential of ventricular myocytes from normal and failing hearts. *Circ Res*. 2003;93:46-53.
12. Mosqueira M, Aykut G and Fink RHA. Mepivacaine reduces calcium transients in isolated murine ventricular cardiomyocytes. *BMC Anesthesiol*. 2020;20:10.
13. Yampolsky P, Koenen M, Mosqueira M, Geschwill P, Nauck S, Witzenberger M, Seyler C, Fink T, Kruska M, Bruehl C, Schwoerer AP, Ehmke H, Fink RHA, Draguhn A, Thomas D, Katus HA and Schweizer PA. Augmentation of myocardial If dysregulates calcium homeostasis and causes adverse cardiac remodeling. *Nat Commun*. 2019;10:3295.
14. Oravecz K, Kormos A, Gruber A, Marton Z, Kohajda Z, Mirzaei L, Jost N, Levijoki J, Pollesello P, Koskelainen T, Otsomaa L, Toth A, Papp JG, Nanasi PP, Antoons G, Varro A, Acsai K and Nagy N. Inotropic effect of NCX inhibition depends on the relative activity of the reverse NCX assessed by a novel inhibitor ORM-10962 on canine ventricular myocytes. *Eur J Pharmacol*. 2018;818:278-286.
15. Otsomaa L, Karjalainen A, Koskelainen T, Levijoki J, Syrjänen K, Wohlfahrt G. (2018). Compounds useful as inhibitors of sodium-calcium exchanger, WO 2019 175464 A1, Orion Corporation, Finland. p. 42 pp.
16. Ruffolo RR, Jr. and Messick K. Systemic hemodynamic effects of dopamine, (+/-)-dobutamine and the (+)-and (-)-enantiomers of dobutamine in anesthetized normotensive rats. *Eur J Pharmacol*. 1985;109:173-81.
